# Design, Synthesis, Binding and Docking-Based 3D-QSAR Studies of 2-Pyridylbenzimidazoles—A New Family of High Affinity CB1 Cannabinoid Ligands

**DOI:** 10.3390/molecules18043972

**Published:** 2013-04-04

**Authors:** Jaime A. Mella-Raipán, Carlos F. Lagos, Gonzalo Recabarren-Gajardo, Christian Espinosa-Bustos, Javier Romero-Parra, Hernán Pessoa-Mahana, Patricio Iturriaga-Vásquez, Carlos David Pessoa-Mahana

**Affiliations:** 1 Departamento de Química y Bioquímica, Facultad de Ciencias, Universidad de Valparaíso, Casilla 5030, Avda. Gran Bretaña 1111, Playa Ancha, Valparaíso, Chile; 2 Departamento de Farmacia, Facultad de Química, Pontificia Universidad Católica de Chile, Casilla 306, 22, Santiago, Chile; 3 Departamento de Química Orgánica y Físicoquímica, Facultad de Química y Ciencias Farmacéuticas, Universidad de Chile, Casilla 233, Santiago, Chile; 4 Departamento de Química, Facultad de Ciencias, Universidad de Chile, Casilla 233, Santiago, Chile

**Keywords:** cannabinoid, CB1 receptor, binding, docking, 3D-QSAR

## Abstract

A series of novel 2-pyridylbenzimidazole derivatives was rationally designed and synthesized based on our previous studies on benzimidazole **14**, a CB1 agonist used as a template for optimization. In the present series, 21 compounds displayed high affinities with K_i_ values in the nanomolar range. **JM-39** (compound **39**) was the most active of the series (K_iCB1_ = 0.53 nM), while compounds **31** and **44** exhibited similar affinities to WIN 55212-2. CoMFA analysis was performed based on the biological data obtained and resulted in a statistically significant CoMFA model with high predictive value (*q^2^* = 0.710, *r^2^* = 0.998, *r^2^_pred_* = 0.823).

## 1. Introduction

**Figure 1:**
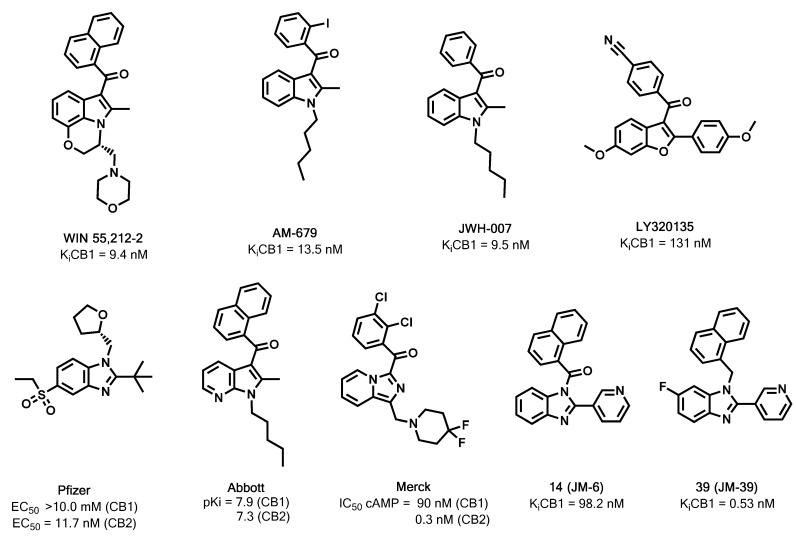
Previously and recently reported cannabinoid ligands, prototype **14** (**JM-6**), and **39** (**JM-39**), the most active compound of the present series. K_i_CB1 data for **14** (**JM-6**) and **39** (**JM-39**) reported herein.

The regulation of the endocannabinoid system (ECS) has been studied extensively because of its great potential in the treatment of many conditions. CB1 agonists has been associated to the treatment of pain, [1], cancer [2], and emesis [3] among others. Otherwise, CB1 antagonists have been mainly used in the treatment of obesity [4,5]. Its physiological effects are mediated through the so-called cannabinoid receptors, which are attractive targets for current drug development [6,7]. These proteins belong to the G protein-coupled receptor (GPCR) family and have been the focus of much recent work since the discovery of the CB1 and CB2 receptor subtypes [8,9]. Among the reported and non-classical cannabinergic ligands [10,11,12], several compounds contain a central heterocyclic scaffold such as indole or benzofuran, displaying a range of different pharmacological profiles [13,14]. For example, the aminoalkylindole derivative WIN55212-2 has been reported to be a potent agonist of the CB1 receptor (K_i_ = 9.4 nM) [15], AM679 is a non-selective CB1 and CB2 agonist (K_i_ = 13.5 nM CB1) [16], *J*WH-007 has been reported as a non selective CB1/CB2 cannabinoid agonist (K_i_ = 9.5 nM) [17], while the benzofuran derivative LY320135 is a selective CB1 antagonist (K_i_ = 131 nM) [18,19] (Figure 1). In the field of cannabimimetic heterocycles, benzimidazoles have been recently reported as CB2 agonist with good CNS penetration by Pfizer [20]. By other hand, Abbott Laboratories have developed azaindoles with agonist activity over the CB1 and CB2 receptors [21], while imidazopyridines from Merck have been reported to be selective CB2 agonists [22] (Figure 1).

For many years our research group has been interested in the synthesis of benzimidazole derivatives as ligands of the type 1 cannabinoid receptor. In recent screening tests using mice brain synaptosomes [23], compound **14** (**JM-6**) was identified as a high affinity CB1 agonist (K_i_ = 98.2 nM). Considering the structural resemblance of **14** to the reported ligands (Figure 1), we defined a strategy to develop new and more active CB1 ligand derivatives. Docking simulation studies of **14** with our reported CB1 model [24] allowed us to propose the existence of three major interaction regions: (a) a hydrogen bond between the pyridine ring and the quaternary amino group of the side chain of residue K3.28(192); (b) the naphthalene moiety establishing hydrophobic and aromatic interactions with the side-chain residues F2.57(170), F2.61(174), L3.32(196), I5.31(267), F7.35(379), A7.36(380); and (c) the benzene ring of the benzimidazole core which displayed hydrophobic and aromatic interactions with the side chain residues F2.57(170), F3.36(200), W6.48(356) and L6.51(359).

**Figure 2:**
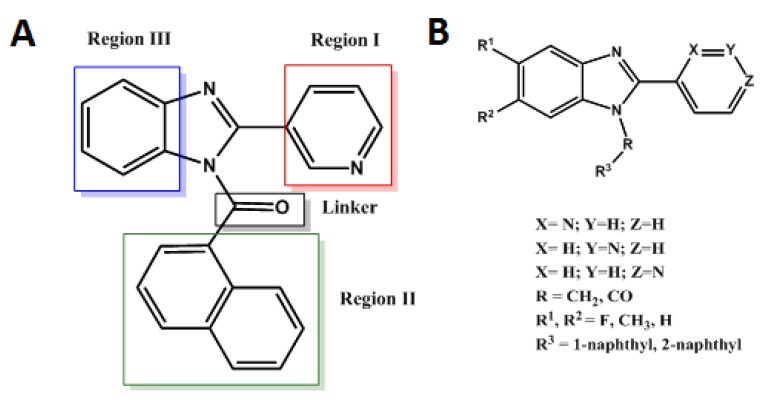
A. Regions for modification on compound **14**. B. General structure of synthesized compounds.

In order to explore SAR requirements of benzimidazoles and optimize the affinity with the CB1 receptor, compound **14** was subjected to the following changes (Figure 2A): (i) Region I: replacement of the 3-pyridyl ring by 2- and 4-pyridyl isomers to study the influence of the position of the nitrogen atom on hydrogen bond interactions. (ii) Region II and linker: the 1-naphthyl moiety was replaced by a 2-naphthyl group and the carbonyl function exchanged for a methylene group aimed at providing an improved orientation and a more favorable conformation in the receptor cavity. (iii) Region III. Finally, substitution on the benzimidazole ring (C-5 and C-6) with fluorine and methyl groups was thought to increase π-stacking interactions. Accordingly, we report the design, synthesis and biological evaluation of novel series of 1 and 2-naphthyl, and 1 and 2-naphthoyl -2-pyridyl-benzimidazole derivatives (Figure 2B) targeting the CB1 receptor. Structure-activity relationships were explored by molecular docking in an updated CB1 receptor model and a 3D-QSAR (CoMFA) was developed using the docking-based alignment.

## 2. Results and Discussion

### 2.1. Chemistry

**Scheme 1:**
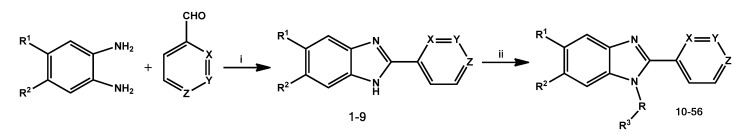
Synthesis of Compounds **1**–**9** and **10**–**56**.

The synthetic sequence for obtaining the target compounds is shown in Scheme 1. A series of substituted *o*-phenylenediamines (benzene-1,2-diamine; 4,5-dimethylbenzene-1,2-diamine and 4-fluorobenzene-1,2-diamine) were reacted with 2-, 3- and 4-pyridinecarbaldehyde in ethanol in the presence of cerium (IV) ammonium nitrate (CAN) and H_2_O_2_ as oxidizing agents [25], yielding benzimidazole derivatives **1**–**9**. The obtained benzimidazoles **1**–**9** were finally acylated [26,27] with 1- or 2-naphthoyl chloride or alkylated with 1- or 2-(bromomethyl)naphthalene to yield compounds **10**–**56**.

*Reagents and Conditions*: (i) Ce(NH_4_)_2_(NO_3_)_6_ (CAN), H_2_O_2_, 50 °C 1 h. (ii) THF, NaH, 1- or 2-naphthoyl chloride, or 1- or 2-(bromomethyl)naphthalene.

**Figure 3:**
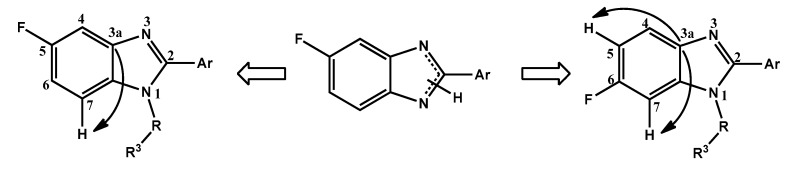
HMBC correlations in regioisomeric products.

Since the benzimidazole ring exists in tautomeric forms, the acylation or alkylation of 2-pyridin-5-fluoro-1*H*-benzo[*d*]imidazoles generate two regioisomers whose structures have been definitively assigned by HMBC experiments which showed an HMBC correlation between C-3a (Figure 3) and the hydrogen at C-7 in the 5-fluoro isomer, and with the hydrogens at C-5 and C-7 in the 6-fluoro isomer.

### 2.2. CB1 Binding Affinities, Docking and SAR

In our efforts to obtain novel CB1 ligands with improved affinities and to gain experimental evidence concerning the mode of interaction of cannabimimetic benzimidazoles with this receptor, we have designed and synthesized a series of benzimidazoles **10**–**56**. The CB1 receptor affinity values for these compounds are shown in Table 1.

**Figure 4:**
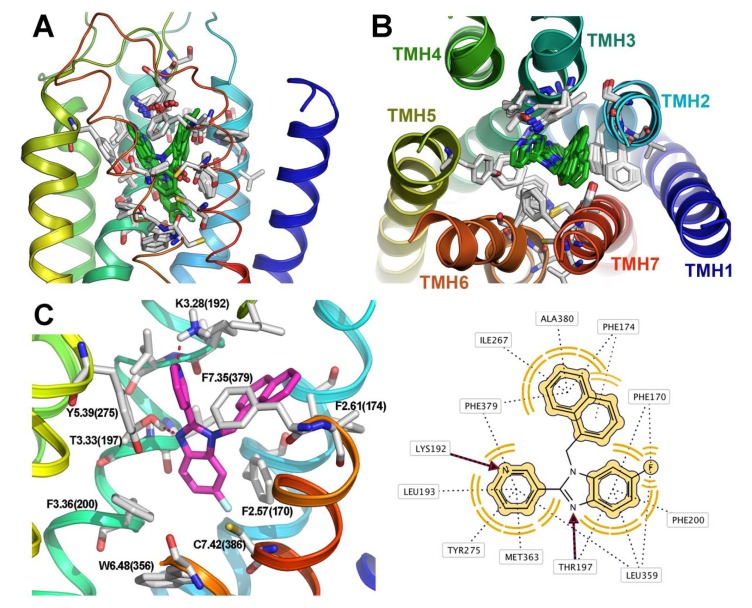
Superimposition of top-scored binding models for the ligands within the CB1 receptor binding site. (**A**) Side view, and (**B**) top view from the extracellular side; the loops have been omitted for greater clarity. (**C**) Schematic representation of the binding mode of the most active compound of the series (**39**). The 2D representation was obtained using LigandScout v3.03. The main interactions are: (1) Two hydrogen bonds, one between the pyridine nitrogen and K3.28(192) and the other between the benzimidazole N-3 and T3.33(197); (2) Hydrophobic and aromatic interactions of the naphthalene ring with the side-chains of F2.61(174), F7.35(379), A7.36 (380), I5.31(267), between the pyridine ring and L3.29(193), Y5.39(275), M6.55(363), F7.35(379) and of the benzimidazole with F2.57(170), F3.36(200), L6.51(359) and W6.48(356).

Significant affinity (nanomolar range) was observed for 21 of the 47 synthesized compounds, while another eight compounds had affinities in the micromolar range. Benzimidazoles **23**, **36** and **51** exhibited IC_50_ values around 20 nM and four molecules (**21**, **31**, **39**, **44**) exhibited an exceptional affinity with IC_50_ values under 10 nM. Docking results showed that all the synthesized compounds adopt a Y-like conformation within the CB1 active site, with the pyridine and naphthalenyl or naphthoyl groups as the arms, and the benzimidazole scaffold as the body (Figure 4A,B). The binding site of the ligands is located amidst transmembrane helices 2, 3, 6 and 7. The binding mode of **39**, the most active compound of the series, is represented in Figure 4C.

**Table 1:** Radioligand displacement results for 2-arylbenzimidazoles **10**–**56** ^a^. 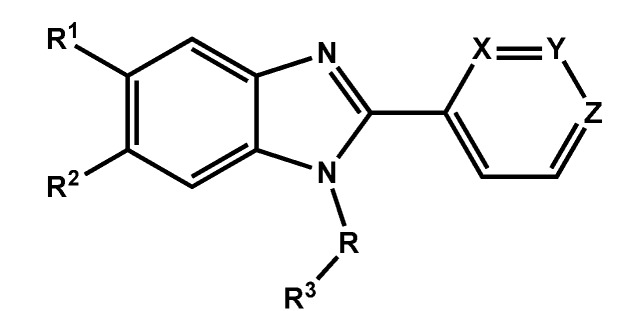

Comp.	X	Y	Z	R	R^1^	R^2^	R^3^	CB1
								(K_i,_ nM)
**10**	N	CH	CH	C=O	H	H	1-naphtyl	n.a
**11**	N	CH	CH	C=O	F	H	1-naphtyl	n.a
**12**	N	CH	CH	C=O	H	F	1-naphtyl	n.a
**13**	N	CH	CH	C=O	CH_3_	CH_3_	1-naphtyl	n.a
**14**	CH	N	CH	C=O	H	H	1-naphtyl	98.24 ± 20.0
**15**	CH	N	CH	C=O	F	H	1-naphtyl	534
**16**	CH	N	CH	C=O	H	F	1-naphtyl	n.a
**17**	CH	N	CH	C=O	CH_3_	CH_3_	1-naphtyl	n.a
**18**	CH	CH	N	C=O	H	H	1-naphtyl	37.81 ± 5.3
**19**	CH	CH	N	C=O	F	H	1-naphtyl	n.a
**20**	CH	CH	N	C=O	H	F	1-naphtyl	n.a
**21**	CH	CH	N	C=O	CH_3_	CH_3_	1-naphtyl	3.95 ± 0.8
**22**	N	CH	CH	C=O	H	H	2-naphtyl	n.a
**23**	N	CH	CH	C=O	H	F	2-naphtyl	11.41 ± 0.9
**24**	N	CH	CH	C=O	CH_3_	CH_3_	2-naphtyl	28.08 ± 2.3
**25**	CH	N	CH	C=O	H	H	2-naphtyl	n.a
**26**	CH	N	CH	C=O	F	H	2-naphtyl	5700 ± 200
**27**	CH	N	CH	C=O	H	F	2-naphtyl	963 ± 155
**28**	CH	N	CH	C=O	CH_3_	CH_3_	2-naphtyl	567 ± 27.8
**29**	CH	CH	N	C=O	H	H	2-naphtyl	4487 ± 133
**30**	CH	CH	N	C=O	F	H	2-naphtyl	1028 ± 57.9
**31**	CH	CH	N	C=O	H	F	2-naphtyl	5.55 ± 0.9
**32**	CH	CH	N	C=O	CH_3_	CH_3_	2-naphtyl	n.a
**33**	N	CH	CH	CH_2_	H	H	1-naphtyl	n.a
**34**	N	CH	CH	CH_2_	F	H	1-naphtyl	n.a
**35**	N	CH	CH	CH_2_	H	F	1-naphtyl	n.a
**36**	N	CH	CH	CH_2_	CH_3_	CH_3_	1-naphtyl	13.04 ± 1.1
**37**	CH	N	CH	CH_2_	H	H	1-naphtyl	75.87 ± 16.6
**38**	CH	N	CH	CH_2_	F	H	1-naphtyl	98.18 ± 33.5
**39**	CH	N	CH	CH_2_	H	F	1-naphtyl	0.53 ± 0.9
**40**	CH	N	CH	CH_2_	CH_3_	CH_3_	1-naphtyl	n.a
**41**	CH	CH	N	CH_2_	H	H	1-naphtyl	149 ± 1.9
**42**	CH	CH	N	CH_2_	F	H	1-naphtyl	n.a
**43**	CH	CH	N	CH_2_	H	F	1-naphtyl	614 ± 54.7
**44**	CH	CH	N	CH_2_	CH_3_	CH_3_	1-naphtyl	4.84 ± 2.0
**45**	N	CH	CH	CH_2_	H	H	2-naphtyl	31.82 ± 12.0
**46**	N	CH	CH	CH_2_	F	H	2-naphtyl	n.a
**47**	N	CH	CH	CH_2_	H	F	2-naphtyl	n.a
**48**	N	CH	CH	CH_2_	CH_3_	CH_3_	2-naphtyl	47.39 ± 11.1
**49**	CH	N	CH	CH_2_	H	H	2-naphtyl	1844 ± 79.8
**50**	CH	N	CH	CH_2_	F	H	2-naphtyl	327 ± 60.0
**51**	CH	N	CH	CH_2_	H	F	2-naphtyl	10.68 ± 2.8
**52**	CH	N	CH	CH_2_	CH_3_	CH_3_	2-naphtyl	140 ± 10.1
**53**	CH	CH	N	CH_2_	H	H	2-naphtyl	464 ± 30.3
**54**	CH	CH	N	CH_2_	F	H	2-naphtyl	44.42 ± 5.5
**55**	CH	CH	N	CH_2_	H	F	2-naphtyl	567 ± 7.0
**56**	CH	CH	N	CH_2_	CH_3_	CH_3_	2-naphtyl	241 ± 10.0
WIN55212								9.40 ^b^
CP55940								0.60 ^b^

*^a^* Data are means ± SEM of n = 3 separate experiments and are expressed as K_i_ (nM). n.a. = compounds which did not displace [^3^H]CP-55,940 by more than 50% at 1 µM in the preliminary screen. ^b^ Ki values obtained from technical data sheet of Perkin Elmer.

In order to obtain useful structure-activity relationships concerning the mode of interaction of the benzimidazole series, we have focused our analysis on the molecular regions defined in Figure 2A.

#### Region I

First, we assessed the effect on affinity of replacing the 3-pyridyl moiety. According to our docking results, this region is crucial for ligand stabilization through an H-bond with the amino group of Lys192. The remainder of the ring presents hydrophobic interactions with the side chains of residues L3.29(193), Y5.39(275) and L6.51(359). Sixteen benzimidazoles were synthesized with a 3-pyridyl group at C-2. Besides the reference compound **14**, four derivatives displayed affinities in the nanomolar range (<200 nM, **39**, **51**, **37**, **38**, Table 1).

As expected, variation of the N position in the pyridinyl substituent to generate the corresponding 2-pyridyl homologs (compounds **35**, **47**, **33**, **34**, **10**) had a dramatic effect on CB1 affinities (Table 1). The inactivity of these compounds might be explained in terms of an unfavorable distance between Lys 192 and the nitrogen to establish a hydrogen bond.

The 4-pyridyl analogs (compounds **43**, **55**, **41**, **42**, **56**) showed modest affinities compared with the 3-pyridyl derivatives (Table 1). Formation of a weaker H-bond between the nitrogen atom and Lys192 compared to the 3-pyridyl compounds seems to be a reasonable explanation. The increased distance between the pyridine nitrogen and Lys192 on going from 3- to 4-pyridyl derivatives observed in our docking models (2 Å and 2.5 Å respectively) corroborates this. However, the activity/inactivity seems actually to be related to the still unknown combined effect of more than one modification. That could explain some exemptions to the observed SAR trends as when homologous compounds **13**, **17** and **21** are compared. In summary, 3- or 4-pyridyl seems to be a better substituent than 2-pyridyl in region I.

#### Region II

According to our docking studies, the naphthyl ring interacts with the CB1 receptor via hydrophobic and aromatic interactions with the side-chains of hydrophobic residues. A comparison of the most active 1-naphthyl derivatives (**39**, **44**, **36**, **37**, **38** and **41**) with their 2-naphthyl congeners (**51**, **56**, **48**, **49**, **50** and **53**) was carried out. In general, the 2-naphthyl derivatives displayed lower CB1 receptor affinities than their 1-naphthyl congeners (Table 1). For benzimidazoles bearing a 2-naphthyl moiety, the naphthalene ring is oriented in a less favourable spatial region in order to interact with the hydrophobic area delimited by Phe379, Phe174, Val196, Ala 380, Ile267 and Phe170.

#### Linker

The methylene bridge does not present any important interaction with the protein, but ligands containing a keto group as linker can establish, according to our docking analysis, hydrogen bonds with Ser383. However, when the carbonyl group of prototype **14** (K_i_ = 98.24 nM) was replaced by a methylene linker (compound **37**, K_i_ = 75.87 nM) no significant variation of CB1 receptor affinity was observed. In fact, derivatives with both carbonyl and methylene are among the most active compounds of the series (Table 1).

There appears to be no *a priori* explanation for this experimental evidence. In order to gain some insight into the origin of these unexpected differences in receptor affinity, a comparative molecular modeling and receptor docking study was carried out for the naphthylmethyl and the corresponding naphthoyl congeners. No significant differences were seen in the angular arrangement of the naphthalene ring and the pose of the benzimidazole was maintained. Discarding a different spatial orientation induced by the linker as the explanation for the loss in affinity, we focused our analysis on the penalty imposed by the ligand desolvation factor. As described in the literature [28], desolvation of the ligand plays a significant role in the binding affinity in the cases where the ligand must be entirely desolvated to perform its function. This is of special relevance in membrane proteins because the ligand has to be transferred from the aqueous environment to the transmembrane binding site. Replacement of the carbonyl bridge by a methylene group could enhance the CB1 receptor binding affinities due to a significant decrease in the desolvation penalty, facilitating the entrance of the ligands into the binding site and compensating for the weaker ligand-receptor interaction. This was confirmed by semiempirical AM1-RHF solvation energy calculations (VAMP software) which displayed an increase in the amount of heat required to raise the entropy of carbonyl compounds.

#### Region III

In order to study the effect of C5 and C6 substitution of the benzimidazole ring on CB1 affinity, the substitution pattern at these positions for the most potent compounds of the series was analyzed. As already mentioned, according to our docking studies the phenyl ring of the benzimidazole core displayed hydrophobic and aromatic interactions with the side chain of residues Phe170, Trp356 and Leu359. Besides, the basic nitrogen of the benzimidazole nucleus forms a hydrogen bond with the side chain of Thr197 for all the compounds.

**Figure 5:**
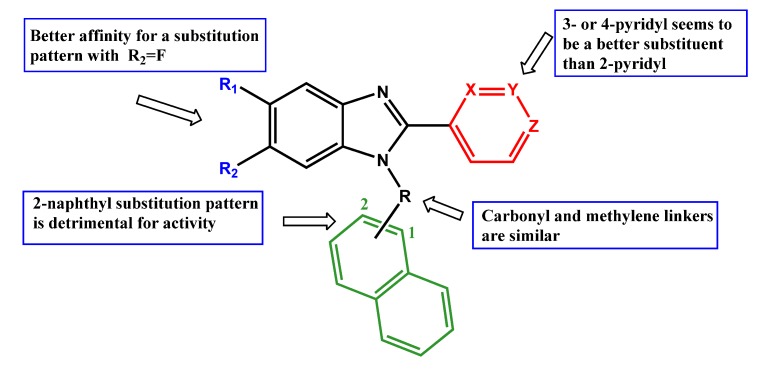
Structure-activity relationships.

In general, substitution at the C5 and C6 positions of the phenyl ring of the benzimidazoles with methyl or fluorine seems to be beneficial for CB1 receptor affinity. Indeed, *J*ust two of the most potent derivatives displayed in Table 1 (compounds **45** and **18**) are not substituted on this ring. The enhancement of π-stacking interactions between the benzimidazole ring and Phe170 and Trp356 is suggested from our docking analysis. The most potent derivatives are monofluorinated at C6 (**39**, **31**, **51**, **23**). On the other hand, an important decrease in affinity is seen when the fluorine is at C5, keeping the rest unchanged (**38**, **30**, **50**). Our docking analysis revealed a possible solvation sphere of water mediating a H bond between the fluorine atom at C6 fluorine and the TM2 Phe170 backbone, which might be crucial to enhance affinity. The structure-activity relationships for the complete series are summarized in Figure 5.

### 2.3. 3D-QSAR (CoMFA)

**Figure 6:**
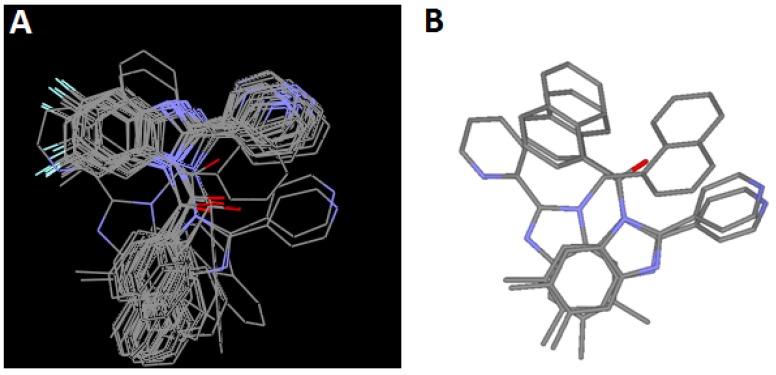
(**A**) Alignment of the 29 studied molecules. (**B**) Docking conformation of compounds **21**, **36** and **44**.

Active conformation selection is a key step for CoMFA analysis [29]. On the other hand, structural alignment is viewed as one of the most sensitive parameters in CoMFA analysis [30]. The predictive accuracy of CoMFA models and the reliability of the contours are directly dependent on the structural alignment rule [30]. In this case this problem was solved using the conformations obtained by docking studies and active compounds were aligned according to the flexible-ligand docking solutions. As can be seen on Figure 6, there are some molecules that are not well overlayed with respect to many of the other derivatives. Specifically, compounds **21**, **36** and **44** display a different docking conformation. Derivatives **21** and **44** are directing the pyridine ring to the same region as the rest of the series compounds. It is noteworthy that compounds **21**, **36** and **44** are dimethylated at C5 and C6 positions of the benzimidazole and localize the dimethylated ring towards the same region where the other compounds of the series direct the naphthalene ring. Indeed, this region on the active site favors hydrophobic interactions with bulky substituents. Finally Gasteiger-Hückel charges [31,32] were assigned to all the molecules.

A 3D-QSAR model was obtained from CoMFA analysis and its statistical parameters are listed in Table 2. For a reliable predictive model, the square *q^2^* of the cross-validation coefficient should be greater than 0.5 [33]. This model has high *r^2^* (0.998), F (944.13), and *q^2^* (0.71), as well as a small SEE (0.057), suggesting that it is reliable and predictive. The steric and electrostatic contributions were found to be 35% and 65% respectively.

**Table 2:** Statistical parameters of the CoMFA model *^a^*.

Statistic Index Summary of CoMFA										
	*N*	*q^2^*	*r^2^*	*r^2^_pred_*	SEE	SD	Press	F	Contribution	
									Steric	Electrostatic
	7	0.71	0.998	0.823	0.057	5.18	0.92	944.1	35	65
**Number of Components Analysis**										
*SEP*	0.781	0.707	0.660	0.614	0.619	0.624	**0.624**	0.649	0.675	0.706
*q^2^*	0.376	0.511	0.594	0.667	0.679	0.692	**0.710**	0.706	0.703	0.698
*N*	1	2	3	4	5	6	**7**	8	9	10

*^a^N* is the optimal number of components, *q^2^* is the square of the LOO cross-validation (CV) coefficient, *r^2^* is the square of non cross-validation coefficient, *r^2^_pred_* is the predictive *r^2^* based only on the test set molecules, SEE is the standard error of estimation of non CV analysis, SEP is the standard error of prediction of LOO analysis, SD is the sum of the squared deviations between the biological activities of molecules in the test set and the mean activity of the training-set molecules, PRESS is the sum of the squared deviations between predicted and actual biological activity values for every molecule in the test set, and F is the F-test value.

**Figure 7:**
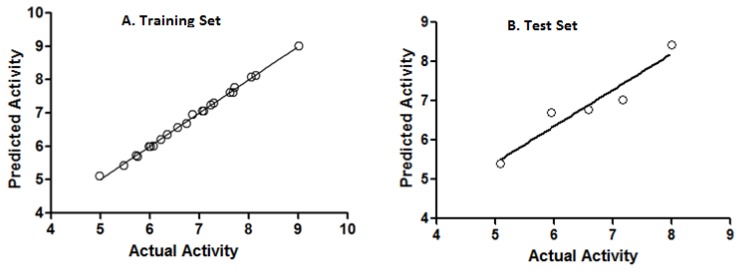
Experimental *versus* predicted activity for A. Training Set and B. Test Set.

**Figure 8:**
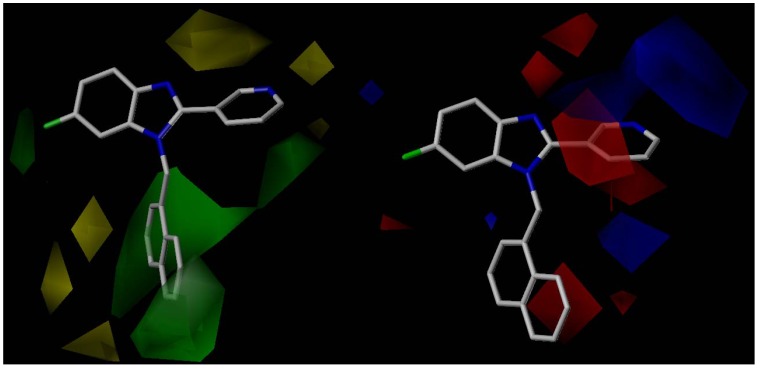
Left: CoMFA steric contour map for the most active compound **39**. The green contour indicates where a bulky group favors activity, while the yellow contour indicates where a small group favors activity. Right: CoMFA electrostatic contour map for the most active compound **39**. The blue contour indicates where positive charge favors activity, while a red contour indicates where negative charge favors activity.

The activity values of compounds predicted by CoMFA are listed in Table 3. The plot of the predicted *p*IC_50_ values versus the experimental ones for CoMFA analysis is also shown in Figure 7, in which most points are evenly distributed along the line *Y* = *X*, suggesting that the quality of the 3D-QSAR model is good. The steric and electrostatic contour maps of CoMFA are displayed in Figure 8.

**Table 3:** Experimental and predicted activity for training and test set.

Compd.	Experimental activity (*p*IC_50_, M)	Predicted activity (*p*IC_50_, M)	Residual
**Training Set**			
**39**	9.009	9.015	−0.006
**21**	8.140	8.126	0.013
**44**	8.052	8.076	−0.024
**51**	7.708	7.767	−0.059
**23**	7.679	7.624	0.056
**36**	7.621	7.622	0.000
**24**	7.288	7.300	−0.011
**45**	7.234	7.237	−0.003
**54**	7.089	7.067	0.022
**48**	7.061	7.067	−0.006
**37**	6.857	6.954	−0.097
**38**	6.745	6.686	0.059
**14**	6.745	6.693	0.052
**41**	6.563	6.575	−0.012
**56**	6.355	6.351	0.004
**50**	6.222	6.208	0.014
**53**	6.070	6.009	0.061
**15**	6.009	6.000	0.008
**28**	5.983	6.005	−0.022
**55**	5.983	5.998	−0.015
**27**	5.753	5.692	0.061
**30**	5.725	5.726	−0.001
**49**	5.471	5.429	0.042
**26**	4.981	5.115	−0.134
**Test Set**			
**31**	7.992	8.433	−0.441
**18**	7.159	7.032	0.128
**52**	6.592	6.777	−0.185
**43**	5.949	6.703	−0.755
**29**	5.085	5.409	−0.325

#### Region I

The CoMFA steric contour map shows two yellow polyhedra near the pyridine ring suggesting that substitution or replacement of this ring with bulky groups is detrimental for activity. An optimal distance for the H-bond with Lys192 is a very important steric restriction in this region. On the other hand, the CoMFA electrostatic contour map displays red areas above and below the pyridine ring, which means that electronegative atoms are required on this ring for optimal activity. This is consistent with the hydrogen bonding between the pyridine nitrogen and the Lys192 residue observed in our docking analysis. Moreover, next to the red areas two blue areas can be seen. A detailed analysis of the active site reveals the presence of amino acids like tyrosine and threonine, available for H-bonding interactions, beyond Lys192, suggesting that replacement of the pyridine with extended hydrogen-bonding donor groups able to reach these residues might also generate active compounds.

#### Linker and Region II

For region II the CoMFA steric contour map shows large green polyhedra around the naphthyl group, suggesting that bulky groups at this position favor affinity. This result is coherent with our docking results, which displayed the naphthyl group oriented toward the lumen of the receptor without any spatial restrictions. With regard to the CoMFA electrostatic contour map, high electron density rings would increase affinity in the region of the naphthyl ring. Not only surfaces provide valuable information, but empty areas too. That is clear analyzing the corresponding CoMFA electrostatic contour map for the linker region, which from the standpoint of the CoMFA seems not to be involved in any H-bonding interaction.

#### Region III

The CoMFA steric contour map reveals a small green surface near the fluorine atom at C6, supporting the synthesis of new compounds substituted at that position, for example with chlorine or bromine. The other positions of the benzimidazole moiety (C4 and C5) do not display any steric restrictions. With respect to the CoMFA electrostatic contour map, the C5 and C6 positions of the benzimidazole ring do not seen to be implicated in relevant electrostatic interactions.

## 3. Experimental

### 3.1. General

All organic solvents used for the syntheses were analytical grade. Melting points were determined on a Stuart Scientific SMP3 apparatus and are uncorrected. IR spectra were recorded on a Bruker Vector 22 spectrophotometer using KBr discs. ^1^H and ^13^C-NMR spectra were obtained on a Bruker AM-400 spectrometer. The chemical shifts are expressed in ppm (δ scale) downfield from TMS, *J* values are given in Hertz for solutions in CDCl_3_ unless otherwise indicated. Column chromatography was performed on Merck silica gel 60 (70–230 mesh). Thin layer chromatographic separations were performed on Merck silica gel 60 (70–230 mesh) chromatofoils. Purity of all final derivatives for biological testing was confirmed to be >95% as determined using elemental analysis carried out on a FISONS EA 1108 CHNS-O analyzer.

#### General Method for 2-Pyridinyl-1*H*-benzo[*d*]imidazoles **1**–**9**

A mixture of an *o*-phenylenediamine (benzene-1,2-diamine; 4,5-dimethylbenzene-1,2-diamine or 4-fluorobenzene-1,2-diamine, 1 mmol), pyridinecarbaldehyde (pyridine-2-carbaldehyde, pyridine-3-carbaldehyde or pyridine-4-carbaldehyde, 1 mmol), H_2_O_2_ (30%, 4 mmol, 0.4 mL), and NH_4_Ce(NO_3_)_6_ (0.1 mmol, 0.0548 g) was heated at 50 °C for 1 h. After completion of the reaction, the reaction mixture was dissolved in EtOH (10 mL) and then poured into ice-water (30 mL). The solid product was filtered, washed with ice-water, and dried. Purification of the crude material by column chromatography on silica gel with dichloromethane as the eluent yielded target compounds **1**–**9** as pale yellow solids.

*2-(Pyridin-2-yl)-1H-benzo[d]imidazole* (**1**). Yield = 95%. Mp. 238–239 °C. ^1^H-NMR (CDCl_3_ + DMSO-*d_6_*) δ 12.50 (s, 1H), 8.59 (d, *J* = 4.3 Hz, 1H), 8.29 (d, *J* = 7.9 Hz, 1H), 7.79 (t, *J* = 7.2 Hz, 1H), 7.63–7.52 (m, 2H), 7.31 (dd, *J_1_* = 6.8, *J_2_*=5.4 Hz, 1H), 7.14 (dd, *J_1_* = 6.0 Hz, *J*_2_=3.1 Hz, 2H). ^13^C-NMR (CDCl_3_ + DMSO-*d_6_*) δ 151.2, 149.3 (2C), 149.0, 137.2 (2C), 124.5 (2C), 122.8 (2C), 121.6 (2C). IR (KBr) 3400, 1430 cm^−1^. Anal. Calcd. For C_12_H_9_N_3_ (MW 195.22): C, 73.83%; H, 4.65%; N, 21.52%. Found: C, 73.50%; H, 4.60%; N, 21.90%.

*5-Fluoro-2-(pyridin-2-yl)-1H-benzo[d]imidazole* (**2**). Yield = 96%. Mp. >300 °C. ^1^H-NMR (CDCl_3_ + DMSO-*d_6_*) δ 12.95 (s, 1H), 8.67 (d, *J* = 4.2 Hz, 1H), 8.35 (d, *J* = 7.8 Hz, 1H), 7.88 (t, *J* = 7.6 Hz, 1H), 7.61 (s, broad, 1H), 7.40 (dd, *J_1_* = 6.5 Hz, *J_2_* = 4.2 Hz, 1H), 7.33 (s, broad, 1H), 6.99 (t, *J* = 8.4 Hz, 1H). ^13^C-NMR (CDCl_3_ + DMSO-*d_6_*) δ 151.9 (d, *J* = 108.2 Hz), 149.2 (2C), 148.7, 137.3 (3C), 124.6 (2C), 121.7, 110.8 (d, *J* = 11.8 Hz), 98.6 (d, *J* = 25.7 Hz). IR (KBr) 3425, 1400 cm^−1^. Anal. Calcd. For C_12_H_8_FN_3_ (MW 213.21): C, 67.60%; H, 3.78%; N, 19.71%. Found: C, 67.55%; H, 3.38%; N, 19.43%.

*5,6-Dimethyl-2-(pyridin-2-yl)-1H-benzo[d]imidazole* (**3**). Yield = 97%. Mp. >300 °C. ^1^H-NMR (CDCl_3_ + DMSO-*d_6_*) δ 11.20 (s, 1H), 8.50 (ddd, *J*_1_ = 0.7 Hz, *J*_2_ = 1.7 Hz, *J*_3_ = 4.8 Hz, 1H), 7.51 (dt, *J*_1_ = 1,7 Hz, *J*_2_ = 7.7 Hz, 1H), 7.29 (dd, *J*_1_ = 4,8 Hz, *J*_2_ = 7.7 Hz, 1H), 6.77 (d, *J*_1_ = 7.7 Hz, 1H), 6.22 (s, 2H), 2.36 (s, 3H, CH_3_), 2.37 (s, 3H, CH_3_). ^13^C-NMR (CDCl_3_ + DMSO-*d_6_*) δ 157.8, 150.1, 149.1 (2C), 136.8, 124.4, 121.5, 120.1, 120.9 (2C), 110.8 (2C), 20.7, 20.4. IR (KBr) 3430, 1444 cm^−1^. Anal. Calcd. For C_14_H_13_N_3_ (MW 223.27): C, 75.31%; H, 5.87%; N, 18.82%. Found: C, 75.91%; H, 5.86%; N, 18.46%.

*2-(Pyridin-3-yl)-1H-benzo[d]imidazole* (**4**). Yield = 85%. Mp: 255–256 °C. ^1^H-NMR (CDCl_3_) δ 12.58–12.70 (s, 1H), 9.43 (dd, *J_1_* = 2.3 Hz, *J*_2_ = 0.8 Hz, 1H), 8.66 (dd, *J_1_* = 4.8 Hz, *J_2_* = 1.5 Hz, 1H), 8.54–8.51 (m, 1H), 7.82–7.76 (m, 1H), 7.48–7.56 (m, 1H), 7.44 (dd, *J_1_* = 8.1 Hz, *J*_2_ = 4.9 Hz, 1H), 7.21–7.31 (m, 2H). ^13^C-NMR (CDCl_3_) δ 150.8, 149.8, 138.2 (2C), 131.3, 126.7, 125.8, 125.5, 120.8 (2C), 113.5 (2C). IR (KBr) 3420, 1446 cm^−1^. Anal. Calcd. For C_12_H_9_N_3_ (MW 195.22): C, 73.83%; H, 4.65%; N, 21.52%. Found: C, 73.51%; H, 4.60%; N, 21.90%.

*5-Fluoro-2-(pyridin-3-yl)-1H-benzo[d]imidazole* (**5**). Yield = 80%. Mp. 221,5–221,7 °C. ^1^H-NMR (CDCl_3_ + DMSO-*d_6_*) *δ* 12.89 (s, 1H), 9.39 (s, 1H), 8.66 (d, *J* = 4.2 Hz, 1H), 8.49 (d, *J* = 7.9 Hz, 1H), 7.82–7.50 (m, 1H), 7.43 (dd, *J_1_* = 7.9, *J_2_* = 4.9 Hz, 1H), 7.37–7.14 (m, 1H), 7.00 (dt, *J_1_* = 9.8 Hz, *J_2_* = 9.5 Hz, *J_3_* = 2.0 Hz, 1H). ^13^C-NMR (CDCl_3_ + DMSO-*d_6_*) *δ* 150.3, 149 (d, *J* = 254.6 Hz), 147.7, 134.0 (2C), 126.4, 126.3, 123.5 (2C), 110.9 (d, *J* = 8.4 Hz), 108.3, 104.2. IR (KBr) 3045, 1450, 707 cm^−1^. Anal. Calcd. For For C_12_H_8_N_3_F (MW 213.21): C, 67.60%; H, 3.78%; N, 19.71%. Found: C, 67.32%; H, 4.02%; N, 19.38%.

*5,6-Dimethyl-2-(pyridin-3-yl)-1H-benzo[d]imidazole* (**6**). Yield = 88%. Mp. 254.8–254.9 °C. ^1^H-NMR (CDCl_3_ + DMSO-*d_6_*) δ 12.63 (s, 1H), 9.36 (d, *J* = 1.7 Hz, 1H), 8.60 (dd, *J_1_* = 4.7 Hz, *J_2_* = 1.3 Hz, 1H), 8.46 (d, *J* = 8.0 Hz, 1H), 7.44 (dd, *J_1_* = 8.0 Hz, *J_2_* = 4.9 Hz, 1H), 7.38 (s, 2H), 2.36 (s, 6H, 2 × CH_3_). ^13^C-NMR (CDCl_3_ + DMSO-*d_6_*) δ 152.6, 150.5, 150.0, 149.7 (2C), 148.0, 147.5, 133.5 (2C), 131.2, 123.4 (2C), 20.1 (2C). IR (KBr) 3067, 1609 cm^−1^. Anal. Calcd. For C_14_H_13_N_3_ (MW 223.27): C, 75.31%; H, 5.87%; N, 18.82%. Found: C, 73.71%; H, 5.86%; N, 18.85%.

*2-(Pyridin-4-yl)-1H-benzo[d]imidazole* (**7**). Yield = 98%. Mp. 207.3–208 °C. ^1^H-NMR (CDCl_3_ + DMSO-*d_6_*) δ 12.74 (s, 1H), 8.73 (d, *J* = 6.0 Hz, 2H), 8.12 (d, *J* = 6.0 Hz, 2H), 7.74–7.59 (m, 2H), 7.28 (dd, *J_1_* = 6.0, *J_2_* = 3.1 Hz, 2H). ^13^C-NMR (CDCl_3_) δ 150.2, 155.0, 149.1 (2C), 141.7 (2C), 137.6 (2C), 123.0 (2C), 120.6 (2C). IR (KBr) 3448, 1610 cm^−1^. Anal. Calcd. For C_12_H_9_N_3_ (MW 195.92): C, 73.83%; H, 4.65%; N, 21.52%. Found: C, 73.77%; H, 4.50%; N, 21.03%.

*5-Fluoro-2-(pyridin-4-yl)-1H-benzo[d]imidazole* (**8**). Yield = 90%. Mp >300. ^1^H-NMR (CDCl_3_ + DMSO-*d_6_*) δ 13.20 (s, 1H), 8.84–8.65 (m, 2H), 8.20–8.08 (m, 2H), 7.68–7.59 (m, 1H), 7.35 (d, *J* = 7.2 Hz, 1H), 7.02 (t, *J* = 9.2 Hz, 1H). ^13^C-NMR (CDCl_3_ + DMSO-*d_6_*) δ 155.3, 150.0 (2C) 142.3 (2C), 125.7, 125.6(2C), 120.3 (2C), 116.2 (d, *J* = 14.4 Hz), 110.8. IR (KBr) 3449, 1612, 832 cm^−1^. Anal. Calcd. For C_12_H_8_FN_3_ (MW 213.21): C, 67.60%; H, 3.78%; N, 19.71%. Found: C, 67.54%; H, 3.39%; N, 19.31%.

*5,6-Dimethyl-2-(pyridin-4-yl)-1H-benzo[d]imidazole* (**9**). Yield = 98%. Mp >300. ^1^H-NMR (CDCl_3_ + DMSO-*d_6_*) δ 8.85–8.60 (m, 2H), 8.22–8.05 (m, 2H), 7.50–7.36 (m, 2H), 2.37 (s, 6H, 2 × CH_3_). ^13^C-NMR (CDCl_3_ + DMSO-*d_6_*) δ 153.0, 150.0(2C), 147.9, 137.8, 131.8, 130.5 (2C), 120.4 (2C), 110.5 (2C), 20.1 (2C). IR (KBr) 3418, 1609 cm^−1^. Anal. Calcd. For C_14_H_13_N_3_ (MW 223.27): C, 75.31%; H, 5.87%; N, 18.82%. Found: C, 75.81%; H, 5.46%; N, 18.36%.

#### General Method for the Synthesis of (2-Pyridinyl-1*H*-benzo[*d*]imidazol-1-yl)(naphthalenyl)methanones **10**–**32** and 2-Pyridinyl-1-(naphthylmethyl)-1*H*-benzo[*d*]imidazoles **33**–**56**

A solution of 2-pyridinyl-1*H*-benzo[*d*]imidazole (1 mmol) in anhydrous THF (50 mL) containing NaH (1 mmol) and the corresponding 1 or 2-naphthoyl chloride (1 mmol), or 1 or 2-(bromomethyl)naphthalene (1 mmol) was stirred at room temperature for 20 min under a nitrogen atmosphere. The solvent was evaporated under reduced pressure, and the residue was purified by column chromatography on silica gel with dichloromethane as eluent to yield target compounds **10**–**56** as pale yellow solids.

*Naphthalen-1-yl(2-(pyridin-2-yl)-1H-benzo[d]imidazol-1-yl)methanone* (**10**). Yield = 77%. Mp. 138.0–138.1 °C. ^1^H-NMR (CDCl_3_) δ 8.80 (d, *J* = 8.6 Hz, 1H), 8.06 (d, *J* = 7.9 Hz, 1H), 7.93 (d, *J* = 7.1 Hz, 1H), 7.88–7.79 (m, 3H), 7.78–7.70 (m, 2H), 7.64–7.52 (m, 2H), 7.42 (pd, *J_1_* = 7.3 Hz, *J_2_* = 1.4 Hz, 2H), 7.20–7.08 (m, 2H), 6.88 (ddd, *J_1_* = 7.5 Hz, *J_2_* = 4.8 Hz, *J_3_* = 1.2 Hz, 1H). ^13^C-NMR (CDCl_3_) δ 169.0, 152.3, 148.4, 148.2, 142.8, 136.4, 135.6, 133.6, 133.3, 132.9, 131.3, 128.3, 128.3, 126.8, 125.9, 125.5, 124.5, 124.0, 123.5, 123.1(2C), 120.5, 112.9. IR (KBr) 1720 cm^−1^. Anal. Calcd. For C_23_H_15_N_3_O (MW 349.38): C, 79.07%; H, 4.33%; N, 12.03%. Found: C, 79.51%; H, 4.35%; N, 12.02%.

*(5-Fluoro-2-(pyridin-2-yl)-1H-benzo[d]imidazol-1-yl)(naphthalen-1-yl)methanone* (**11**). Yield = 70%. Mp. 167.2–167.9 °C. ^1^H-NMR (CDCl_3_) δ 8.76 (d, *J* = 8.5 Hz, 1H), 8.03 (t, *J* = 7.0 Hz, 1H), 7.85–7.79 (m, 3H), 7.77–7.69 (m, 2H), 7.64–7.54 (m, 3H), 7.19 (dt, *J_1_* = 9.1, *J_2_* = 2.5 Hz, 1H), 7.16–7.10 (m, 2H), 6.88 (dd, *J_1_* = 7.7 Hz, *J_2_* = 4.1 Hz, 1H). ^13^C-NMR (CDCl_3_) δ 168.9, 160.6 (d, *J* = 241.0 Hz), 153.8, 148.4, 148.2, 136.7, 133.7 (d, *J* = 7.8 Hz), 132.9, 132.2, 131.5, 128.5, 128.4, 127.1, 126.0, 124.1, 123.9, 123.4, 121.6 (d, *J* = 10.4 Hz), 114.0, 113.8, 113.7, 106.6 (d, *J* = 24.0 Hz), 100.2 (d, *J* = 28.9 Hz). IR (KBr) 1735 cm^−1^. Anal. Calcd. For C_23_H_14_FN_3_O (MW 367.38): C, 75.19%; H, 3.84%; N, 11.44%. Found: C, 75.59%; H, 4.30%; N, 11.04%.

*(6-Fluoro-2-(pyridin-2-yl)-1H-benzo[d]imidazol-1-yl)(naphthalen-1-yl)methanone* (**12**). Yield = 64%. Mp. 177.2–178.0 °C. ^1^H-NMR (CDCl_3_) δ 8.76 (d, *J* = 8.6 Hz, 1H), 8.02 (d, *J* = 7.6 Hz, 1H), 7.86 (dd, *J_1_* = 9.0 Hz, *J_2_* = 5.1 Hz 1H), 7.84–7.79 (m, 3H), 7.74 (t, *J* = 7.7 Hz, 1H), 7.64–7.52 (m, 2H), 7.48 (dd, *J_1_* = 8.8 Hz, *J_2_* = 2.4 Hz, 1H), 7.19 (dt, *J_1_* = 9.2 Hz, *J_2_* = 2.5 Hz, 1H), 7.17–7.12 (m, 2H), 6.89 (*J_1_* = 7.0 Hz, *J_2_* = 4.7 Hz, 1H). ^13^C-NMR (CDCl_3_) δ 168.7, 161.1 (d, *J* = 243.5 Hz), 152.8, 148.2 (d, *J* = 19.1 Hz), 139.1, 136.5, 133.6, 133.6 (d, *J* = 5.5 Hz), 131.3, 128.4, 128.2, 126.9, 125.8, 123.9, 123.6, 122.9, 121.4, 121.3,113.8, 113.6, 113.0 (d, *J* = 25.3 Hz), 110.0, 100.0 (d, *J* = 29.2 Hz). IR (KBr) 1740 cm^−1^. Anal. Calcd. For C_23_H_14_FN_3_O (MW 367.38): C, 75.19%; H, 3.84%; N, 11.44%. Found: C, 75.49%; H, 4.35%; N, 11.94%.

*(5,6-Dimethyl-2-(pyridin-2-yl)-1H-benzo[d]imidazol-1-yl)(naphthalen-1-yl)methanone* (**13**). Yield = 93%. Mp. 167.2–168.0 °C. ^1^H-NMR (CDCl_3_) δ 8.63 (d, *J* = 4.5 Hz, 1H), 8.57 (d, *J* = 4.3 Hz, 1H), 8.47 (d, *J* = 8.0 Hz, 1H), 7.84 (td, *J_1_* = 7.7 Hz, *J_2_* = 1.8 Hz, 1H), 7.65 (s broad, 1H), 7.51 (td, *J_1_* = 7.7 Hz, *J_2_* = 1.8 Hz, 1H), 7.36–7.26 (m, 2H), 7.22–7.11 (m, 2H), 6.87 (d, *J* = 7.9 Hz, 1H), 6.28 (s, 2H), 2.41 (s, 3H, CH_3_), 2.37 (s, 3H, CH_3_). ^13^C-NMR (CDCl_3_) δ 158.0, 150.9, 149.3, 148.8, 144.0, 141.6, 139.6, 137.1, 137.0, 135.7, 133.4, 132.2, 131.6(2C), 128.7, 126.5, 125.7, 125.0, 124.5, 123.8, 114.7, 113.1, 110.9, 18.8(2C). IR (KBr) 1635 cm^−1^. Anal. Calcd. For C_25_H_19_N_3_O (MW 377.44): C, 79.55%; H, 5.07%; N, 11.13%; Found: C, 79.49%; H, 5.35%; N, 11.54%.

*Naphthalen-1-yl-(2-(pyridin-3-yl)-1H-benzo[d]imidazol-1-yl)methanone* (**14**). Yield = 79%. Mp. 148.0–148.1 °C. ^1^H-NMR (CDCl_3_) δ 8.67 (s, 1H), 8.33 (s, 1H), 7.98 (d, *J* = 7.97 Hz, 1H), 7.88 (d, *J* = 3.65 Hz, 1H), 7.86 (d, *J* = 3.98 Hz, 1H), 7.80 (d, *J* = 7.20 Hz, 1H), 7.66 (d, *J* = 8.10 Hz, 1H), 7.60 (d, *J* = 7.27 Hz, 2H), 7.54 (t, *J_1_* = 7.08 Hz, *J_2_* = 7.08 Hz, 2H), 7.42 (t, *J_1_* = 7.58 Hz, *J_2_* = 7.58 Hz, 1H), 7.38–7.28 (m, 2H), 6.90 (s, 1H). ^13^C-NMR (CDCl_3_) δ 151.4, 150.1, 149.2, 142.9, 135.7, 133.9 (2C), 133.5, 130.7 (2C), 130.1 (2C), 128.7, 127.1, 125.7, 125.2, 124.5, 124.2 (2C), 122.17, 120.6, 114.2 (2C). IR (KBr) 1708 cm^−1^. Anal. Calcd. For C_23_H_15_N_3_O (MW 349.38): C, 79.07%; H, 4.33%; N, 12.03%. Found: C, 79.64%; H, 4.34%; N, 12.12%.

*(5-Fluoro-2-(pyridin-3-yl)-1H-benzo[d]imidazol-1-yl)(naphthalen-1-yl)methanone* (**15**). Yield = 60%. Mp. 169.7–170.7 °C. ^1^H-NMR (CDCl_3_) δ 8.60 (d, *J* = 1.3 Hz, 1H), 8.30 (dd, *J_1_* = 1.3 Hz, *J_2_* = 4.9 Hz 1H), 7.99 (d, *J* = 8.2 Hz, 1H), 7.87 (d, *J* = 8.2 Hz, 1H), 7.81 (d, *J* = 7.6 Hz, 1H), 7.71 (dd, *J_1_* = 9.0 Hz, *J_2_* = 4.7 Hz, 1H), 7.63–7.52 (m, 5H), 7.31 (t, *J* = 7.7 Hz, 1H), 7.13 (dt, *J_1_* = 9.1 Hz, *J_2_* = 2.4 Hz, 1H), 6.87 (dd, *J_1_* = 7.7 Hz, *J_2_* = 4.9 Hz, 1H).^13^C-NMR (CDCl_3_) δ 168.2, 160.8 (d, *J* = 242.1 Hz), 152.9, 150.4, 149.3, 143.7 (d, *J* = 12.4 Hz), 139.3, 135.8, 134.2, 133.6, 131.2, 130.7 (d, *J* = 21.6 Hz), 130.2, 128.97, 129.0, 128.3, 126.9, 124.6, 124.2, 122.3, 115.2 (d, *J* = 9.5 Hz), 113.9 (d, *J* = 25.5 Hz), 106.7 (d, *J* = 24.4 Hz). IR (KBr) 1735 cm^−1^. Anal. Calcd. For C_23_H_14_FN_3_O (MW 367.38): C, 75.19%; H, 3.84%; N, 11.44%. Found: C, 75.19%; H, 3.35%; N, 11.34%.

*(6-Fluoro-2-(pyridin-3-yl)-1H-benzo[d]imidazol-1-yl)(naphthalen-1-yl)methanone* (**16**). Yield = 55%. Mp. 168.1–168.7 °C. ^1^H-NMR (CDCl_3_) δ 8.60 (d, *J* = 1.6 Hz, 1H), 8.29 (dd, *J_1_* = 4.9 Hz, *J_2_* = 1.7 Hz, 1H), 7.99 (d, *J* = 8.5 Hz, 1H), 7.88 (d, *J* = 8.2 Hz, 1H), 7.85–7.79 (m, 2H) 7.63–7.53 (m, 4H), 7.49 (dd, *J_1_* = 9.0 Hz, *J_2_* = 2.4 Hz, 1H), 7.33 (dd, *J_1_* = 8.2 Hz, *J_2_* = 7.3 Hz, 1H), 7.20 (td, *J_2_* = 9.0 Hz, *J_2_* = 2.5 Hz, 1H), 6.87 (dd, *J_1_* = 7.8 Hz, *J_2_* = 4.9 Hz, 1H).^13^C-NMR (CDCl_3_) δ 168.3, 161.2 (d, *J* = 243.6 Hz), 151.9, 151.9, 150.2, 149.2, 139.4, 135.8, 135.0 (d, *J* = 13.8 Hz), 134.3, 133.6, 130.8, 130.5, 130.3, 129.0 (d, *J* = 3.1 Hz), 127.4, 127.0, 124.61, 124.2, 122.4, 121.5 (d, *J* = 10.1 Hz), 113.7 (d, *J* = 24.9 Hz), 101.9 (d, *J* = 29.4 Hz). IR (KBr) 1740 cm^−1^ Anal. Calcd. For C_23_H_14_FN_3_O (MW 367.38): C, 75.19%; H, 3.84%; N, 11.44%. Found: C, 75.20%; H, 3.95%; N, 11.92%.

*(5,6-Dimethyl-2-(pyridin-3-yl)-1H-benzo[d]imidazol-1-yl)(naphthalen-1-yl)methanone* (**17**). Yield = 73%. Mp. 177.2–178.0 °C. ^1^H-NMR (CDCl_3_/DMSO-*d_6_*) δ 8.51 (s, 1H), 8.25 (s, 1H), 8.03–7.95 (m, 1H), 7.95–7.88 (m, 2H), 7.74–7.66 (m, 2H), 7.65–7.51 (m, 3H), 7.50–7.45 (m, 1H), 7.39 (t, *J* = 7.72 Hz, 1H), 7.02–6.89 (m, 1H), 2.39 (s, 3H, CH_3_), 2.32 (s, 3H, CH_3_). ^13^C-NMR (CDCl_3_/DMSO-*d_6_*) δ 188.8, 168.3, 150.7, 150.0, 149.0, 141.5, 136.2, 134.7, 134.0, 133.6, 133.3, 133.0, 131.0, 130.3, 129.0, 128.7, 127.4, 127.2, 125.0, 124.3, 122.5, 120.5, 114.6, 20.7, 20.3. IR (KBr) 1637 cm^−1^. Anal. Calcd. For C_25_H_19_N_3_O (MW 377.44): C, 79.55%; H, 5.07%; N, 11.13%; Found: C, 80.00%; H, 5.57%; N, 11.62%.

*Naphthalen-1-yl-(2-(pyridin-4-yl)-1H-benzo[d]imidazol-1-yl)methanone* (**18**). Yield = 65%. Mp. 155.0–156.1 °C. ^1^H-NMR (DMSO-*d_6_*) δ 7.55–7.43 (m, 3H), 7.22–7.07 (m, 3H), 6.97–6.90 (m, 2H), 6.88–6.83 (m, 1H), 6.82–6.76 (m, 1H), 6.72–6.65 (m, 1H), 6.65–6.53 (m, 2H), 6.52–6.46 (m, 2H). ^13^C-NMR (CDCl_3_) δ 166.6, 150.7(2C), 148.2, 142.3, 137.1, 134.3, 133.0, 132.7, 130.5, 130.4, 129.3, 128.0, 127.8, 126.2, 124.8, 124.1, 124.0, 123.4, 121.8(2C), 120.2, 113.3. IR (KBr) 1720 cm^−1^. Anal. Calcd. For C_23_H_15_N_3_O (MW 349.38): C, 79.07%; H, 4.33%; N, 12.03%. Found: C, 79.04%; H, 4.84%; N, 12.10%.

*(5-Fluoro-2-(pyridin-4-yl)-1H-benzo[d]imidazol-1-yl)(naphthalen-1-yl)methanone* (**19**). Yield = 62%. Mp. 180.7–181.7°C. ^1^H-NMR (CDCl_3_) δ 8.30–8.26 (m, 2H), 8.11 (d, *J* = 8.3 Hz, 1H), 7.90 (d, *J* = 8.2 Hz, 1H), 7.83 (d, *J* = 7.7 Hz, 1H), 7.69 (dd, *J_1_* = 9.0 Hz, *J_2_* = 4.7 Hz, 1H), 7.63 (dt, *J_1_* = 8.5 Hz, *J_2_* = 1.6 Hz, 1H), 7.60–7.55 (m, 2H), 7.54 (d, *J* = 7.0 Hz, 1H), 7.29 (dd, *J_1_* = 7.6 Hz, *J_2_* = 7.9 Hz 1H), 7.26–7.23 (m, 2H), 7.15 (td, *J_1_* = 9.1 Hz, *J_2_* = 2.5 Hz, 1H). ^13^C-NMR (CDCl_3_) δ 168.0, 160.8 (d, *J* = 242.8 Hz), 153.1, 149.1, 143.5 (d, *J* = 12.4 Hz), 138.3, 134.6, 133.6, 133.5, 131.3, 131.0, 130.5, 130.4, 129.1(2C), 127.8, 126.3, 124.3 (d, *J* = 12.1 Hz), 122.9(2C), 115.2 (d, *J* = 9.6 Hz), 114.4 (d, *J* = 25.4 Hz), 107.0 (d, *J* = 24.5 Hz). IR (KBr) 1710 cm^−1^. Anal. Calcd. For C_23_H_14_FN_3_O (MW 367.38): C, 75.19%; H, 3.84%; N, 11.44%. Found: C, 75.69%; H, 4.25%; N, 11.92%.

*(6-Fluoro-2-(pyridin-4-yl)-1H-benzo[d]imidazol-1-yl)(naphthalen-1-yl)methanone* (**20**). Yield = 63%. Mp. 190.4–190.9 °C. ^1^H-NMR (CDCl_3_) δ 8.29–8.24 (m, 2H), 8.12 (d, *J* = 8.4 Hz, 1H), 7.91 (d, *J* = 8.5 Hz, 1H), 7.87–7.81 (m, 2H), 7.67–7.57 (m, 2H), 7.54 (dd, *J_1_* = 7.2 Hz, *J_2_* = 1.3 Hz, 1H) 7.47 (dd, *J_1_* = 8.9 Hz, *J_2_* = 2.5 Hz, 1H), 7.30 (dd, *J_1_* = 8.3 Hz, *J_2_* = 7.2 Hz, 1H), 7.25–7.21 (m, 2H), 7.19 (dd, *J_1_* = 9.0 Hz, *J_2_* = 2.5 Hz, 1H).^13^C-NMR (CDCl_3_) δ 168.0, 161.4 (d, *J* = 244.4 Hz), 152.2 (d, *J* = 3.6 Hz), 150.5, 149.2(2C), 139.3, 138.2, 135.2, 135.1, 134.7, 133.6, 131.0, 130.4, 130.4, 129.1, 127.4, 124.3 (d, *J* = 12.4 Hz), 122.8(2C), 121.8 (d, *J* = 10.2 Hz), 113.9 (d, *J* = 25.0 Hz), 101.8 (d, *J* = 29.4 Hz). IR (KBr) 1733 cm^−1^. Anal. Calcd. For C_23_H_14_FN_3_O (MW 367.38): C, 75.19%; H, 3.84%; N, 11.44%. Found: C, 74.98%; H, 4.33%; N, 11.01%. 

*(5,6-Dimethyl-2-(pyridin-4-yl)-1H-benzo[d]imidazol-1-yl)(naphthalen-1-yl)methanone* (**21**). Yield = 77%. Mp. 167.2–168.0 °C. ^1^H-NMR (CDCl_3_) δ 8.20 (d, *J* = 5.9 Hz, 2H), 8.07 (d, *J* = 8.3 Hz, 1H), 7.86 (d, *J* = 8.2 Hz, 1H), 7.80 (d, *J* = 7.6 Hz, 1H), 7.64 (s, 1H), 7.62–7.48 (m, 4H), 7.30–7.23 (m, 1H), 7.17 (d, *J* = 5.9 Hz, 2H), 2.41 (s, 3H, CH_3_), 2.36 (s, 3H, CH_3_).^13^C-NMR (CDCl_3_) δ 168.5, 151.1, 149.3(2C), 141.5, 138.6, 135.8, 134.6, 134.2, 133.6, 133.4, 131.2, 131.1, 130.3, 129.0, 128.8, 127.2, 124.5, 124.4, 122.8(2C), 120.9, 114.7, 20.9, 20.5. IR (KBr) 1617 cm^−1^. Anal. Calcd. For C_25_H_19_N_3_O (MW 377.44): C, 79.55%; H, 5.07%; N, 11.13%; Found: C, 79.05%; H, 5.52%; N, 10.98%.

*Naphthalen-2-yl(2-(pyridin-2-yl)-1H-benzo[d]imidazol-1-yl)methanone* (**22**). Yield = 82%. Mp. 145.0–146.0 °C. ^1^H-NMR (CDCl_3_) δ 8.29 (d, *J* = 8.0 Hz, 1H), 8.22 (d, *J* = 4.7 Hz, 1H), 8.05 (s, 1H), 7.97–7.91 (m, 2H), 7.86–7.81 (m, 2H), 7.74 (d, *J* = 8.2 Hz, 1H), 7.69 (td, *J_1_* = 7.7 Hz, *J_2_* = 1.7 Hz, 1H), 7.56 (td, *J_1_* = 8.1 Hz, *J_2_* = 1.2 Hz, 1H), 7.53–7.46 (m, 2H), 7.41 (td, *J_1_* = 8.1 Hz, *J_2_* = 7.7 Hz, *J_3_* = 1.3 Hz, 1H), 7.35 (d, *J* = 8.2 Hz, 1H), 7.05 (ddd, *J_1_* = 7.6 Hz, *J_2_* = 4.8 Hz, *J_3_* = 1.2 Hz, 1H). ^13^C-NMR (CDCl_3_) δ 169.4, 152.0, 148.5, 148.1, 143.0, 136.7, 135.6, 135.5, 132.2, 131.8, 131.5, 129.5, 128.9, 128.8, 127.8, 127.0, 125.3, 125.1, 124.2, 124.0, 123.3, 120.5, 112.3. IR (KBr) 1715 cm^−1^. Anal. Calcd. For C_23_H_15_N_3_O (MW 349.38): C, 79.07%; H, 4.33%; N, 12.03%. Found: C, 79..57%; H, 4.83%; N, 12.01%.

*(6-Fluoro-2-(pyridin-2-yl)-1H-benzo[d]imidazol-1-yl)(naphthalen-2-yl)methanone* (**23**). Yield = 66%. Mp. 195.7–195.9 °C. ^1^H-NMR (CDCl_3_) δ 8.78 (s, 1H), 8.61 (d, *J* = 4.8 Hz, 1H), 8.54 (d, *J* = 7.9 Hz, 1H), 8.21 (d, *J* = 8.6 Hz, 1H), 8.00 (d, *J* = 8.1 Hz, 1H), 7.92 (t, *J* = 9.0 Hz, 3H), 7.66 (dd, *J_1_* = 8.7 Hz, *J_2_* = 4.7 Hz, 1H),7.63–7.53 (m, 2H), 7.45–7.34 (m, 2H), 7.06 (td, *J_1_* = 9.2 Hz, *J_2_* = 2.4 Hz, 1H). ^13^C-NMR (CDCl_3_) δ 171.0, 160.1 (d, *J* = 239.6 Hz), 151.3, 148.6, 147.8, 139.0, 138.1, 135.8, 132.6, 131.8, 129.5, 128.4 (d, *J* = 18.9 Hz), 127.8, 127.6, 127.4, 126.7, 125.7, 124.9, 123.2, 122.6, 116.8, 112.1 (d, *J* = 25.5 Hz), 101.6 (d, *J* = 40.1 Hz). IR (KBr) 1723 cm^−1^. Anal. Calcd. For C_23_H_14_FN_3_O (MW 367,38): C, 75.19%; H, 3.84%; N, 11.44%. Found: C, 75.20%; H, 4.26%; N, 11.00%. 

*(5,6-Dimethyl-2-(pyridin-2-yl)-1H-benzo[d]imidazol-1-yl)(naphthalen-2-yl)methanone* (**24**). Yield = 85%. Mp. 168.7–169.6 °C. ^1^H-NMR (CDCl_3_) δ 8.59 (d, *J* = 4.9 Hz, 1H), 8.53 (d, *J* = 5.0 Hz, 1H), 8.45 (d, *J* = 8.0 Hz, 1H), 7.81 (td, *J*_1_ = 7.7 Hz, *J*_2_ = 1.8 Hz, 1H), 7.62 (s, 1H), 7.48 (td, *J*_1_ = 7.7 Hz, *J*_2_ = 1.9 Hz, 1H), 7.31–7.22 (m, 2H), 7.19–7.04 (m, 2H), 6.84 (d, *J* = 7.8 Hz, 1H), 6.25 (s, 2H), 2.38 (s, 3H, CH_3_ ), 2.33 (s, 3H, CH_3_). ^13^C-NMR (CDCl_3_) δ 157.8, 153.1, 150.6, 149.1, 148.6, 148.5, 147.9, 147.8, 141.4, 139.0, 136.9, 136.8, 135.4, 133.2, 132.0, 124.3, 123.5, 122.2, 120.8, 120.1, 119.0, 118.4, 110.7, 20.6, 20.3. IR (KBr) 1629 cm^−1^. Anal. Calcd. For C_25_H_19_N_3_O (MW 377.44): C, 79.55%; H, 5.07%; N, 11.13%; Found: C, 80.04%; H, 5.57%; N, 10.84%. 

*Naphthalen-2-yl(2-(pyridin-3-yl)-1H-benzo[d]imidazol-1-yl)methanone* (**25**). Yield = 90%. Mp. 148.0–148.6 °C. ^1^H-NMR (CDCl_3_) δ 8.92 (s, 1H), 8.46 (d, *J* = 3.3 Hz, 1H), 8.27 (s, 1H), 7.97 (dt, *J*_1_ = 7.9 Hz, *J*_2_ = 1.8 Hz, 1H), 7.93 (d, *J* = 8.0 Hz, 1H), 7.90–7.86 (m, 2H), 7.85–7.80 (m, 2H), 7.65 (dt, *J*_1_ = 7.6 Hz, *J*_2_ = 1.1 Hz, 1H), 7.56 (dt, *J*_1_ = 7.5 Hz, *J*_2_ = 1.0 Hz, 1H), 7.41 (dt, *J*_1_ = 7.6 Hz, *J*_2_ = 1.2 Hz, 1H), 7.36 (d, *J* = 7.9 Hz, 1H), 7.31–7.26 (m, 1H), 7.19 (dd, *J*_1_ = 7.7 Hz, *J*_2_ = 4.9 Hz, 1H). ^13^C-NMR (CDCl_3_) δ 168.7, 151.4, 150.5, 149.7, 143.0, 136.2, 136.0, 135.0, 133.1, 132.2, 129.8, 129.6, 129.5, 129.3, 128.0, 127.5, 127.0, 125.3, 125.2, 124.8, 123.0, 120.6, 113.3. IR (KBr) 1755 cm^−1^. Anal. Calcd. For C_23_H_15_N_3_O (MW 349.38): C, 79.07%; H, 4.33%; N, 12.03%. Found: C, 78..61%; H, 4.87%; N, 12.02%.

*(5-Fluoro-2-(pyridin-3-yl)-1H-benzo[d]imidazol-1-yl)(naphthalen-2-yl)methanone* (**26**). Yield = 50%. Mp. 177.7–118.6 °C. ^1^H-NMR (CDCl_3_) δ 8.63 (s, 1H), 8.51 (d, *J* = 8.1 Hz, 1H), 8.25 (s, 1H), 8.09 (d, *J* = 8.6 Hz, 1H), 7.95 (d, *J* = 8.0 Hz, 1H), 7.91–7.82 (m, 3H), 7.79 (dd, *J_1_* = 8.6 Hz, *J_2_* = 1.8 Hz, 1H), 7.63–7.51 (m, 3H), 7.35 (dd, *J_1_* = 9.0 Hz, *J_2_* = 4.6 Hz, 1H), 7.07 (dt, *J_1_* = 9.1 Hz, *J_2_* = 2.5 Hz, 1H). ^13^C-NMR (CDCl_3_) δ 168.3, 160.2 (d, *J* = 241.6 Hz), 152.7, 150.5, 150.4, 149.5, 147.9, 143.5 (d, *J* = 12.1 Hz), 136.2, 135.9, 135.4, 133.0, 132.0, 131.4, 129.7, 129.4 (d, *J* = 3.8 Hz), 128.0, 127.6, 126.5, 125.0, 114.0 (d, *J* = 9.7 Hz), 113.2 (d, *J* = 26.0 Hz), 106.4 (d, *J* = 23.6 Hz). IR (KBr) 1715 cm^−1^. Anal. Calcd. For C_23_H_14_FN_3_O (MW 367.38): C, 75.19%; H, 3.84%; N, 11.44%. Found: C, 74.89%; H, 4.27%; N, 11.42%. 

*(6-Fluoro-2-(pyridin-3-yl)-1H-benzo[d]imidazol-1-yl)(naphthalen-2-yl)methanone* (**27**). Yield = 50%. Mp. 167.2–167.7 °C. ^1^H-NMR (CDCl_3_) δ 8.74 (s, 1H), 8.46 (d, *J* = 8.5 Hz, 1H), 8.26 (s, 1H), 8.17 (d, *J* = 8.6 Hz, 1H), 7.98 (d, *J* = 8.4 Hz, 1H), 7.95–7.76 (m, 3H), 7.70–7.54 (m, 2H), 7.49 (dd, *J_1_* = 8.8 Hz, *J_2_* = 4.6 Hz, 1H), 7.24–7.11 (m, 2H), 6.96 (t, *J* = 8.3 Hz, 1H). ^13^C-NMR (CDCl_3_) δ 168.4, 160.4 (d, *J* = 158.9 Hz), 151.6, 150.3, 149.4, 139.3, 136.4, 136.0, 135.7, 135.3, 134.9, 132.1, 129.4 (d, *J* = 10.9 Hz), 129.3, 128.4, 128.0, 127.8, 127.7, 126.7, 125.1, 121.4 (d, *J* = 10.2 Hz), 113.2 (d, *J* = 25.4 Hz), 111.9 (d, *J* = 25.4 Hz). IR (KBr) 1739 cm^−1^. Anal. Calcd. For C_23_H_14_FN_3_O (MW 367.38): C, 75.19%; H, 3.84%; N, 11.44%. Found: C, 74.70%; H, 4.34%; N, 11.93%.

*(5,6-Dimethyl-2-(pyridin-3-yl)-1H-benzo[d]imidazol-1-yl)(naphthalen-2-yl)methanone* (**28**). Yield = 75%. Mp. 166.7–166.9 °C. ^1^H-NMR (CDCl_3_) δ 8.98 (s broad, 1H), 8.51 (s broad, 1H), 8.32 (s, 1H), 8.02 (dt, *J*_1_ = 7.9 Hz, *J*_2_ = 1.9 Hz, 1H), 7.96 (d, *J* = 8.5 Hz, 2H), 7.94–7.87 (m, 2H), 7.77 (s, 1H), 7.73 (td, *J*_1_ = 7.6 Hz, *J*_2_ = 1.4 Hz, 1H), 7.65 (td, *J*_1_ = 7.5 Hz, *J*_2_ = 1.2 Hz, 1H), 7.34 (s, 1H), 7.24 (d, *J* = 5.2 Hz, 1H), 2.51 (s, 3H, CH_3_ ), 2.40 (s, 3H, CH_3_). ^13^C-NMR (CDCl_3_) δ 169.1, 150.3, 149.8, 148.1, 144.0, 141.7, 136.2, 136.0, 134.9, 134.0, 133.7, 133.2, 132.3, 130.2, 129.6(2C), 129.3, 128.1, 127.6, 125.5, 123.1, 120.7, 113.7, 20.8, 20.5. IR (KBr) 1639 cm^−1^. Anal. Calcd. For C_25_H_19_N_3_O (MW 377.44): C, 79.55%; H, 5.07%; N, 11.13%; Found: C, 80.04%; H, 5.56%; N, 10.64%. 

*Naphthalen-2-yl(2-(pyridin-4-yl)-1H-benzo[d]imidazol-1-yl)methanone* (**29**). Yield = 75%. Mp. 145.0–146.1 °C. ^1^H-NMR (CDCl_3_) δ 8.57 (s broad, 2H), 8.27 (s, 1H), 7.93 (d, *J* = 8.3 Hz, 1H), 7.90 (s broad, 1H), 7.88-–7.86 (m, 1H), 7.83 (d, *J* = 8.1 Hz, 1H), 7.66 (t, *J* = 7.2 Hz, 1H), 7.61–7.53 (m, 3H), 7.42 (t, *J* = 7.4 Hz, 1H), 7.35–7.26 (m, 2H), 7.88–7.86 (m, 1H). ^13^C-NMR (CDCl_3_) δ 168.7, 151.8, 150.1, 143.1, 138.2, 136.2, 135.3, 133.3, 132.3, 129.9, 129.9, 129.6, 129.5, 128.2, 127.8(2C), 125.7, 125.4(2C), 125.0, 123.2, 121.0, 113.4. IR (KBr) 1731 cm^−1^. Anal. Calcd. For C_23_H_15_N_3_O (MW 349.38): C, 79.07%; H, 4.33%; N, 12.03%. Found: C, 79.57%; H, 4.33%; N, 12.01%.

*(5-Fluoro-2-(pyridin-4-yl)-1H-benzo[d]imidazol-1-yl)(naphthalen-2-yl)methanone* (**30**). Yield = 40%. Mp. 195.7–200.6 °C. ^1^H-NMR (CDCl_3_) δ 8.52 (d, *J* = 5.5 Hz, 2H), 8.22 (s, 1H), 7.88 (t, *J* = 8.4 Hz, 2H), 7.84–7.79 (m, 2H), 7.65 (t, *J* = 7.5 Hz, 1H), 7.59 (d, *J_1_* = 8.35 Hz, *J_2_* = 2.5 Hz, 1H), 7.57–7.55 (m, 1H), 7.53 (d, *J* = 5.5 Hz, 2H), 7.31 (dd, *J_1_* = 9.0 Hz, *J_2_* = 4.6 Hz, 1H), 7.05 (td, *J_1_* = 9.1 Hz, *J_2_* = 2.5 Hz, 1H). ^13^C-NMR (CDCl_3_) δ 168.4, 160.5 (d, *J* = 242.0 Hz), 153.1, 150.0(2C), 143.6 (d, *J* = 12.6 Hz), 137.9, 136.2, 133.2, 132.1, 131.7, 130.0, 129.5(3C), 128.1, 127.8, 125.2, 123.1(2C), 114.1, 113.9 (d, *J* = 26.2 Hz), 106.8 (d, *J* = 24.4 Hz). IR (KBr) 1755 cm^−1^. Anal. Calcd. For C_23_H_14_FN_3_O (MW 367.38): C, 75.19%; H, 3.84%; N, 11.44%. Found: C, 75.69%; H, 3.37%; N, 11.40%. 

*(6-Fluoro-2-(pyridin-4-yl)-1H-benzo[d]imidazol-1-yl)(naphthalen-2-yl)methanone* (**31**). Yield = 55%. Mp. 187.3–188.2°C. ^1^H-NMR (CDCl_3_) δ 8.52 (d, *J* = 6.1 Hz, 2H), 8.23 (s, 1H), 7.95–7.80 (m, 5H), 7.67 (t, *J* = 7.6 Hz, 1H), 7.59 (d, *J* = 7.6 Hz, 1H), 7.53 (d, *J* = 6.1 Hz, 2H), 7.18 (td, *J_1_* = 9.0 Hz, *J_2_* = 2.4 Hz, 1H), 7.12 (dd, *J_1_* = 9.0 Hz, *J_2_* = 2.5 Hz, 1H).^13^C-NMR (CDCl_3_) δ 170.3, 161.6 (d, *J* = 245.0 Hz), 159.3, 152.2, 150.0 (2C), 143.7 (d, *J* = 11.9 Hz), 143.0, 138.0, 136.0, 133.2, 132.2, 131.7, 130.1, 129.5, 128.0, 127.8, 125.2, 123.0(2C), 114.0, 113.8 (d, *J* = 26.2 Hz), 106.7 (d, *J* = 24.3 Hz). IR (KBr) 1754 cm^−1^. Anal. Calcd. For C_23_H_14_FN_3_O (MW 367.38): C, 75.19%; H, 3.84%; N, 11.44%. Found: C, 75.01%; H, 3.94%; N, 11.43%.

*(5,6-Dimethyl-2-(pyridin-4-yl)-1H-benzo[d]imidazol-1-yl)(naphthalen-2-yl)methanone* (**32**). Yield = 85%. Mp. 160.4–160.9 °C. ^1^H-NMR (CDCl_3_) δ 8.49 (s broad, 1H), 8.21 (s, 1H), 7.94–7.77 (m, 4H), 7.70–7.64 (m, 2H), 7.60 (dd, *J*_1_ = 3.8 Hz, *J*_2_ = 1.4 Hz, 1H), 7.57–7.48 (m, 3H), 7.20 (s, 1H), 2.41 (s, 3H, CH_3_), 2.29 (s, 3H, CH_3_). ^13^C-NMR (CDCl_3_) δ 168.8, 149.8, 141.5, 138.3, 136.0, 135.2, 134.0, 133.0(2C), 132.0, 129.9, 129.6, 129.4(2C), 129.2, 128.0(2C), 127.5, 125.2, 120.7, 120.0, 113.4, 113.4, 20.7, 20.3. IR (KBr) 1616 cm^−1^. Anal. Calcd. For C_25_H_19_N_3_O (MW 377.44): C, 79.55%; H, 5.07%; N, 11.13%; Found: C, 79.54%; H, 5.49%; N, 11.62%. 

*1-(Naphthalen-1-ylmethyl)-2-(pyridin-2-yl)-1H-benzo[d]imidazole* (**33**). Yield: 70%. Mp. 150.7–151.6 °C. ^1^H-NMR (CDCl_3_) δ 8.48 (d, *J* = 8.0 Hz, 1H), 8.44 (d, *J* = 4.2 Hz, 1H), 8.19 (d, *J* = 8.4 Hz, 1H), 7.95 (d, *J* = 8.1 Hz, 1H), 7.91 (d, *J* = 8.1 Hz, 1H), 7.80 (td, *J*_1_ = 7.8 Hz, *J*_2_ = 1.4 Hz, 1H), 7.72 (d, *J* = 8.2 Hz, 1H), 7.63 (t, *J* = 7.2 Hz, 1H), 7.57 (t, *J* = 7.3 Hz, 1H), 7.37–7.32 (m, 1H), 7.26–7.17 (m, 4H), 6.70 (s, 2H, CH_2_), 6.63 (d, *J* = 7.1 Hz, 1H). ^13^C-NMR (CDCl_3_) δ 150.5, 148.7, 142.8, 139.9, 137.1, 136.8, 133.6, 132.8, 130.5, 129.0, 127.6, 126.4, 125.9, 125.6, 124.5, 123.8, 123.7, 123.0, 122.9, 122.5, 120.3, 110.8, 46.8. IR (KBr) 1447 cm^−1^. Anal. Calcd. For C_23_H_17_N_3_ (MW 335.40): C, 82.36%; H, 5.11%; N, 12.53%; Found: C, 82.86%; H, 5.10%; N, 12.07%.

*5-Fluoro-1-(naphthalen-1-ylmethyl)-2-(pyridin-2-yl)-1H-benzo[d]imidazole* (**34**). Yield: 55%. Mp. 155.7–155.9 °C. ^1^H-NMR (CDCl_3_) δ 8.50–8.41 (m, 2H), 8.16 (d, *J* = 8.3 Hz, 1H), 7.91 (d, *J* = 7.9 Hz, 1H), 7.81 (td, *J*_1_ = 7.7 Hz, *J*_2_ = 1.8 Hz, 1H), 7.73 (d, *J* = 8.1 Hz, 1H), 7.64–7.54 (m, 3H), 7.27–7.19 (m, 2H), 7.10 (dd, *J*_1_ = 8.9 Hz, *J*_2_ = 4.6 Hz, 1H), 6.96 (td, *J*_1_ = 9.1 Hz, *J*_2_ = 2.4 Hz, 1H), 6.68 (s, 2H, CH_2_), 6.61 (d, *J* = 7.2 Hz, 1H). ^13^C-NMR (CDCl_3_) δ 159.8 (d, *J* = 238.2 Hz), 151.7, 150.2, 148.8, 143.1 (d, *J* = 12.5 Hz), 136.9, 133.6, 132.5, 130.5, 129.0, 127.8, 127.7, 126.5, 126.0, 125.6, 124.6, 124.0, 122.9, 122.4, 114.0, 112.2 (d, *J* = 26.3 Hz), 111.2 (d, *J* = 10.2 Hz), 105.7 (d, *J* = 24.0 Hz), 47.0. IR (KBr) 1456 cm^−1^. Anal. Calcd. For C_23_H_16_FN_3_ (MW 353.39): C, 78.17%; H, 4.56%; N, 11.89% Found: C, 78.07%; H, 5.01%; N, 12.02%.

*6-Fluoro-1-(naphthalen-1-ylmethyl)-2-(pyridin-2-yl)-1H-benzo[d]imidazole* (**35**). Yield = 50%. Mp. 160.7–161.1 °C. ^1^H-NMR (CDCl_3_) δ 8.49–8.40 (m, 2H), 8.16 (d, *J* = 8.3 Hz, 1H), 7.92 (d, *J* = 8.0 Hz, 1H),7.84 (dd, *J*_1_ = 9.0 Hz, *J*_2_ = 4.9 Hz, 1H), 7.79 (dt, *J*_1_ = 8.0 Hz, *J*_2_ = 1.6 Hz, 1H), 7.74 (d, *J* = 8.2 Hz, 1H), 7.63 (t, *J*_2_ = 7.7 Hz, *J*_2_ = 1.5 Hz, 1H), 7.57 (t, *J* = 7.2 Hz, 1H), 7.28–7.19 (m, 2H), 7.07 (td, *J*_1_ = 9.2 Hz, *J*_2_ = 2.4 Hz, 1H), 6.88 (dd, *J*_1_ = 8.8 Hz, *J*_2_ = 2.5 Hz, 1H), 6.65 (s, 2H, CH_2_), 6.63 (d, *J* = 6.9 Hz, 1H). ^13^C-NMR (CDCl_3_) δ 160.3 (d, *J* = 241.5 Hz), 151.2, 150.3, 148.8, 139.2, 137.4 (d, *J* = 13.4 Hz), 136.9, 133.7, 132.3, 130.5, 129.0, 127.8, 126.5, 126.0, 125.6, 124.3, 123.9, 122.9, 122.42, 121.1 (d, *J* = 10.3 Hz), 111.6 (d, *J* = 25.1 Hz), 97.4 (d, *J* = 27.3 Hz), 47.1. IR (KBr) cm^−1^: 1446. Anal. Calcd. For C_23_H_16_FN_3_ (MW 353.39): C, 78.17%; H, 4.56%; N, 11.89%. Found: C, 78.56%; H, 4.58%; N, 11.42%.

*5,6-Dimethyl-1-(naphthalen-1-ylmethyl)-2-(pyridin-2-yl)-1H-benzo[d]imidazole* (**36**). Yield = 85%. Mp. 155.5-156.1 °C. ^1^H-NMR (CDCl_3_) δ 8.44 (d, *J* = 7.8 Hz, 1H), 8.40 (d, *J* = 3.8 Hz, 1H), 8.19 (d, *J* = 8.4 Hz, 1H), 7.92 (d, *J* = 7.5 Hz, 1H), 7.77 (t, *J* = 7.8 Hz, 1H), 7.73-7.67 (m, 2H), 7.67-7.61 (m, 1H), 7.60-7.55 (m, 1H), 7.23-7.16 (m, 2H), 6.99 (s, 1H), 6.65 (s, 2H, CH_2_), 6.58 (d, *J* = 7.1 Hz, 1H), 2.40 (s, 3H, CH_3_), 2.28 (s, 3H, CH_3_). ^13^C-NMR (CDCl_3_) δ 150.7, 148.6, 141.5, 136.7, 135.7, 133.6, 133.2, 133.0, 132.0, 130.5, 129.0, 127.4, 126.3, 125.8, 125.7, 124.3, 123.5, 122.7, 122.5, 120.9, 120.2, 110.7, 46.7, 20.6, 20.4. IR (KBr) 1446 cm^−1^. Anal. Calcd. For C_25_H_21_N_3_ (MW 363.45): C, 82.61%; H, 5.82%; N, 11.56%. Found: C, 82.21%; H, 6.31%; N, 11.55%. 

*1-(Naphthalen-1-ylmethyl)-2-(pyridin-3-yl)-1H-benzo[d]imidazole* (**37**). Yield = 80%. Mp. 160.2–161.1 °C. ^1^H-NMR (CDCl_3_) δ 8.88–8.86 (s, 1H), 8.58 (d, *J* = 4.8 Hz, 1H), 7.94 (dt, *J*_1_ = 8.0 Hz, *J*_2_ = 1.6 Hz, 1H), 7.91–7.84 (m, 3H), 7.77 (d, *J* = 8.3 Hz, 1H), 7.56–7.50 (m, 2H), 7.33–7.16 (m, 4H), 7.12 (d, *J* = 7.9 Hz, 1H), 6.79 (d, *J* = 7.1 Hz, 1H), 5.84 (s, 2H, CH_2_). ^13^C-NMR (CDCl_3_) δ 151.3, 150.9, 149.6, 143.3, 136.4, 136.4, 133.8, 130.9, 130, 129.3, 128.6, 127.0, 126.4, 126.3, 125.7, 123.8, 123.6, 123.2, 122.9, 121.9, 120.3, 110.6, 46.6. IR (KBr) 1450 cm^−1^. Anal. Calcd. For C_23_H_17_N_3_ (MW 335.40): C, 82.36%; H, 5.11%; N, 12.53%. Found: C, 82.16%; H, 5.60%; N, 12.67%. 

*5-Fluoro-1-(naphthalen-1-ylmethyl)-2-(pyridin-3-yl)-1H-benzo[d]imidazole* (**38**). Yield = 58%. Mp. 163.4–163.9 °C. ^1^H-NMR (CDCl_3_) δ 8.94 (d, *J* = 0.01Hz, 1H), 8.66 (dd, *J*_1_ = 4.9 Hz, *J*_2_ = 1.7 Hz, 1H), 8.04–7.96 (m, 2H), 7.95–7.91 (m, 1H), 7.86 (d, *J* = 8.3 Hz, 1H), 7.64–7.58 (m, 3H), 7.40–7.30 (m, 2H), 7.10 (dd, *J*_1_ = 8.9 Hz, *J*_2_ = 4.6 Hz, 1H), 7.01 (td, *J*_1_ = 9.1, *J*_2_ = 2.4 Hz, 1H), 6.85 (dd, *J*_1_ = 7.2 Hz, *J*_2_ = 1.3 Hz, 1H), 5.91 (s, 2H, CH_2_). ^13^C-NMR (CDCl_3_) δ 159.9 (d, *J* = 238.7 Hz), 152.7, 151.0, 149.5, 143.7 (d, *J* = 13.0 Hz), 136.3, 133.9, 132.9, 130.6, 129.3, 128.8, 127.8, 127.0, 126.5, 125.7, 123.7, 122.8, 121.8, 114.1, 112.2 (d, *J* = 26.1 Hz), 111.1 (d, *J* = 10.4 Hz), 106.0 (d, *J* = 24.0 Hz), 46.8. IR (KBr) 1476 cm^−1^. Anal. Calcd. For C_23_H_16_FN_3_ (MW 353.39): C, 78.17%; H, 4.56%; N, 11.89%. Found: C, 78.27%; H, 5.05%; N, 12.32%. 

*6-Fluoro-1-(naphthalen-1-ylmethyl)-2-(pyridin-3-yl)-1H-benzo[d]imidazole* (**39**). Yield = 56%. Mp. 156.1–157.0 °C. ^1^H-NMR (CDCl_3_) δ 8.93 (s, 1H), 8.64 (dd, *J*_1_ = 4.9 Hz, *J*_2_ = 1.7 Hz, 1H), 8.01–7.94 (m, 2H), 7.93–7.89 (m, 1H), 7.89–7.82 (m, 2H), 7.64–7.57 (m, 2H), 7.35 (t, *J* = 7.7 Hz, 1H), 7.30 (dd, *J*_1_ = 8.0 Hz, *J*_2_ = 4.9 Hz, 1H), 7.09 (dt, *J*_1_ = 9.2 Hz, *J*_2_ = 2.4 Hz, 1H), 6.89–6.84 (m, 2H), 5.86 (s, 2H, CH_2_). ^13^C-NMR (CDCl_3_) δ 160.2 (d, *J* = 241.8 Hz), 156.4, 152.0 (d, *J* = 3.5 Hz), 150.9, 149.5, 139.7, 136.7 (d, *J* = 13.0 Hz), 136.2, 133.9, 130.4, 129.3, 128.8, 127.0, 126.5, 125.6, 123.6, 122.8, 121.8, 121.2 (d, *J* = 10.0 Hz), 114.1, 111.7 (d, *J* = 25.1 Hz), 97.3 (d, *J* = 27.8 Hz), 46.8. IR (KBr) 1480 cm^−1^. Anal. Calcd. For For C_23_H_16_FN_3_ (MW 353.39): C, 78.17%; H, 4.56%; N, 11.89%. Found: C, 78.61%; H, 4.44%; N, 11.79%. 

*5,6-Dimethyl-1-(naphthalen-1-ylmethyl)-2-(pyridin-3-yl)-1H-benzo[d]imidazole* (**40**). Yield = 90%. Mp. 141.4–142.1 °C. ^1^H-NMR (CDCl_3_) δ 8.88 (s, 1H), 8.59 (d, *J* = 4.4 Hz, 1H), 7.99–7.90 (m, 3H), 7.83 (d, *J* = 8.2 Hz, 1H), 7.69 (s, 1H), 7.64–7.57 (m, 2H), 7.32 (t, *J* = 7.7 Hz, 1H), 7.27–7.21 (m, 1H), 6.94 (s, 1H), 6.85 (d, *J* = 7.1 Hz, 1H), 5.81 (s, 2H, CH_2_), 2.40 (s, 3H, CH_3_), 2.27 (s, 3H, CH_3_). ^13^C-NMR (CDCl_3_) δ 150.5, 150.4, 149.4, 141.9, 136.2, 135.1, 133.8, 133.2, 132.2, 131.2, 130.0, 129.2, 128.5, 126.9, 126.5, 126.4, 125.8, 123.5, 122.9, 122.0, 120.2, 110.6, 46.5, 20.6, 20.4. IR (KBr) 1440 cm^−1^. Anal. Calcd. For C_25_H_21_N_3_ (MW 363.45): C, 82.61%; H, 5.82%; N, 11.56%. Found: C, 83.11%; H, 5.92%; N, 11.57%. 

*1-(Naphthalen-1-ylmethyl)-2-(pyridin-4-yl)-1H-benzo[d]imidazole* (**41**). Yield = 93%. Mp. 163.3–163.8 °C. ^1^H-NMR (CDCl_3_) δ 8.56 (d, *J* = 6.1 Hz, 2H), 7.97–7.88 (m, 3H), 7.81 (d, *J* = 8.3 Hz, 1H), 7.62–7.54 (m, 4H), 7.33 (t, *J* = 7.6 Hz, 1H), 7.28–7.24 (m, 1H), 7.22–7.16 (m, 1H), 7.11 (d, *J* = 8.1 Hz, 1H), 6.81 (d, *J* = 7.1 Hz, 1H), 5.84 (s, 2H, CH_2_). ^13^C-NMR (CDCl_3_) δ 151.3, 150.5(2C), 143.2, 137.5, 136.6, 133.9, 130.9, 129.9, 129.4, 129.3, 128.7, 126.5, 125.7, 124.1, 123.6, 123.4, 122.9(2C), 121.9, 120.6, 110.7, 46.6. IR (KBr) 1449 cm^−1^. Anal. Calcd. For C_23_H_17_N_3_ (MW 335.40): C, 82.36%; H, 5.11%; N, 12.53%. Found: C, 82.86%; H, 5.20%; N, 12.77%. 

*5-Fluoro-1-(naphthalen-1-ylmethyl)-2-(pyridin-4-yl)-1H-benzo[d]imidazole* (**42**). Yield = 53%. Mp. 151.5-152.5 °C. ^1^H-NMR (DMSO-d6) δ 8.65 (d, *J* = 5.2 Hz, 2H), 8.05–7.93 (m, 2H), 7.89 (d, *J* = 8.4 Hz, 1H), 7.70–7.55 (m, 5H), 7.38 (t, *J* = 7.7 Hz, 1H), 7.12 (dd, *J*_1_ = 8.8 Hz, *J*_2_ = 4.6 Hz, 1H), 7.03 (td, *J*_1_ = 8.9 Hz, *J*_2_ = 2.4 Hz, 1H), 6.86 (d, *J* = 7.2 Hz, 1H), 5.93 (s, 2H, CH_2_). ^13^C-NMR (CDCl_3_) δ 160.0 (d, *J* = 238.6 Hz), 152.7, 150.6(2C), 143.6 (d, *J* = 12.8 Hz), 137.2, 133.9, 133.1, 130.5, 129.9, 129.4, 128.9, 127.8 (d, *J* = 5.1 Hz), 127.1, 126.6, 125.7, 122.8(2C), 121.8, 112.7 (d, *J* = 26.3 Hz), 111.2 (d, *J* = 10.0 Hz), 106.2 (d, *J* = 24.2 Hz), 46.8. IR (KBr) 1471 cm^−1^. Anal. Calcd. For C_23_H_16_FN_3_ (MW 353.39): C, 78.17%; H, 4.56%; N, 11.89%. Found: C, 78.67%; H, 4.55%; N, 12.30%. 

*6-Fluoro-1-(naphthalen-1-ylmethyl)-2-(pyridin-4-yl)-1H-benzo[d]imidazole* (**43**). Yield = 60%. Mp. 167.4–168.1°C. ^1^H-NMR (CDCl_3_) δ 8.63 (d, *J* = 5.6 Hz, 2H), 8.04–7.96 (m, 1H), 7.98–7.91 (m, 1H), 7.91–7.84 (m, 2H), 7.74–7.45 (m, 4H), 7.38 (t, *J* = 7.7 Hz, 1H), 7.12 (td, *J*_1_ = 8.9 Hz, *J*_2_ = 2.4 Hz, 1H), 6.91–6.84 (m, 2H), 5.89 (s, 2H, CH_2_). ^13^C-NMR (CDCl_3_) δ 160.4 (d, *J* = 241.6 Hz), 152.0, 150.5(2C), 139.6, 137.2, 133.9, 130.3, 129.9, 129.4, 128.9, 127.8 (d, *J* = 6.7 Hz), 127.1, 126.6, 125.7, 123.1, 122.8(2C), 121.8, 121.5 (d, *J* = 9.8 Hz), 112.0 (d, *J* = 25.3 Hz), 97.3 (d, *J* = 27.5 Hz), 46.8. IR (KBr) 1431 cm^−1^. Anal. Calcd. For C_23_H_16_FN_3_ (MW 353.39): C, 78.17%; H, 4.56%; N, 11.89%. Found: C, 78.38%; H, 5.05%; N, 12.21%. 

*5,6-Dimethyl-1-(naphthalen-1-ylmethyl)-2-(pyridin-4-yl)-1H-benzo[d]imidazole* (**44**). Yield = 92%. Mp. 156.8–157.6 °C. ^1^H-NMR (CDCl_3_) δ 8.59 (s broad, 2H), 7.98 (s broad, 2H), 7.86 (d, *J* = 7.0 Hz, 1H), 7.74–7.53 (m, 5H), 7.39–7.31 (m, 1H), 6.96 (s, 1H), 6.86 (d, *J* = 6.8 Hz, 1H), 5.87 (s, 2H, CH_2_), 2.41 (s, 3H, CH_3_ ), 2.29 (s, 3H, CH_3_ ). ^13^C-NMR (CDCl_3_) δ 150.4(2C), 141.9, 137.7, 135.3, 133.9, 133.7, 132.5, 131.1, 129.9, 128.6, 127.9, 127.0, 126.5, 125.8, 123.1, 122.9, 122.8(2C), 122.0, 120.5, 110.6, 46.6, 20.6, 20.4. IR (KBr) 1455 cm^−1^. Anal. Calcd. For C_25_H_21_N_3_ (MW 363.45): C, 82.61%; H, 5.82%; N, 11.56%. Found: C, 83.07%; H, 6.30%; N, 12.01%. 

*1-(Naphthalen-2-ylmethyl)-2-(pyridin-2-yl)-1H-benzo[d]imidazole* (**45**). Yield = 87%. Mp. 150.7–151.8 °C. ^1^H-NMR (CDCl_3_) δ 8.74 (d, *J* = 4.2 Hz, 1H), 8.59 (d, *J* = 8.0 Hz, 1H), 8.01 (d, *J* = 7.9 Hz, 1H), 7.95 (td, *J* = 7.9, 1.7 Hz, 1H), 7.89 (dd, *J*_1_ = 6.0 Hz, *J*_2_ = 3.4 Hz, 1H), 7.86 (d, *J* = 8.6 Hz, 1H), 7.80 (dd, *J*_1_ = 6.0 Hz, *J*_2_ = 3.4 Hz, 1H), 7.67 (s, 1H), 7.56–7.51 (m, 2H), 7.51-7.45 (m, 2H), 7.44–7.40 (m, 2H), 7.37 (d, *J* = 6.6 Hz, 1H), 6.48 (s, 2H, CH_2_).^13^C-NMR (CDCl_3_) δ 150.6, 150.1, 148.7, 142.8, 136.9, 136.9, 135.0, 133.3, 132.7, 128.5, 127.8, 127.7, 126.2, 125.9, 125.4, 124.9, 124.7, 123.9, 123.7, 122.9, 120.2, 110.8, 49.2. IR (KBr) 1466 cm^−1^. Anal. Calcd. For C_23_H_17_N_3_ (MW 335.40): C, 82.36%; H, 5.11%; N, 12.53%. Found: C, 82.06%; H, 5.61%; N, 12.05%.

*5-Fluoro-1-(naphthalen-2-ylmethyl)-2-(pyridin-2-yl)-1H-benzo[d]imidazole* (**46**). Yield = 62%. Mp. 165.7–166.4 °C. ^1^H-NMR (CDCl_3_) δ 8.65 (d, *J* = 3.9 Hz, 1H), 8.48 (d, *J* = 8.0 Hz, 1H), 7.87 (td, *J*_1_ = 7.9 Hz, *J*_2_ = 1.9 Hz, 1H), 7.83–7.76 (m, 2H), 7.72 (dd, *J*_1_ = 6.2 Hz, *J*_2_ = 3.3 Hz, 1H), 7.61–7.54 (m, 2H), 7.46 (d, *J* = 6.3 Hz, 1H), 7.45 (d, *J* = 6.2 Hz, 1H), 7.39–7.32 (m, 2H), 7.29 (dd, *J*_1_ = 9.0 Hz, *J*_2_ = 4.4 Hz, 1H), 7.02 (td, *J*_1_ = 9.1 Hz, *J*_2_ = 2.4 Hz, 1H), 6.37 (s, 2H, CH_2_). ^13^C-NMR (CDCl_3_) δ 159.8 (d, *J* = 238.1 Hz), 151.3, 150.3, 148.8, 143.2, 143.1, 137.0, 134.7, 133.4, 133.3, 132.8, 128.6, 127.8 (d, *J* = 9.7 Hz), 126.3, 126.0, 125.8, 125.4, 124.8 (d, *J* = 2.8 Hz), 124.1, 112.1 (d, *J* = 26.4 Hz), 111.3 (d, *J* = 10.2 Hz), 105.7 (d, *J* = 23.9 Hz), 49.4. IR (KBr) 1484 cm^−1^. Anal. Calcd. For C_23_H_16_FN_3_ (MW 353.39): C, 78.17%; H, 4.56%; N, 11.89%. Found: C, 77.95%; H, 5.02%; N, 12.35%.

*6-Fluoro-1-(naphthalen-2-ylmethyl)-2-(pyridin-2-yl)-1H-benzo[d]imidazole* (**47**). Yield = 55%. Mp. 170.5–171.3 °C. ^1^H-NMR (CDCl_3_) δ 8.65 (d, *J* = 5.8 Hz, 1H), 8.46 (d, *J* = 8.0 Hz, 1H), 7.9–7.76 (m, 4H), 7.73 (dd, *J*_1_ = 6.2 Hz, *J*_2_ = 3.4 Hz, 1H), 7.58 (s, 1H), 7.47 (d, *J* = 6.3 Hz, 1H), 7.46 (d, *J* = 6.2 Hz, 1H), 7.40–7.31 (m, 2H), 7.13–7.02 (m, 2H), 6.34 (s, 2H, CH_2_). ^13^C-NMR (CDCl_3_) δ 160.3 (d, *J* = 211.9 Hz), 150.4, 148.7, 145.1, 139.2, 137.2, 137.0, 134.5, 133.3, 132.8, 128.6, 128.1, 127.8 (d, *J* = 12.1 Hz), 126.3, 126.0, 125.5, 124.8, 124.5, 124.0, 121.0 (d, *J* = 9.8 Hz), 111.5 (d, *J* = 25.1 Hz), 97.4 (d, *J* = 27.9 Hz), 49.4. IR (KBr) 1464 cm^−1^. Anal. Calcd. For C_23_H_16_FN_3_ (MW 353.39): C, 78.17%; H, 4.56%; N, 11.89%. Found: C, 78.15%; H, 4.52%; N, 12.33%.

*5,6-Dimethyl-1-(naphthalen-2-ylmethyl)-2-(pyridin-2-yl)-1H-benzo[d]imidazole* (**48**). Yield = 77%. Mp. 147.7–148.5 °C. ^1^H-NMR (CDCl_3_) δ 8.61 (d, *J* = 4.3 Hz, 1H), 8.45 (d, *J* = 7.9 Hz, 1H), 7.89–7.75 (m, 3H), 7.67 (s, 1H), 7.58–7.49 (m, 2H), 7.48–7.41 (m, 2H), 7.36 (d, *J* = 8.5 Hz, 1H), 7.31–7.26 (m, 1H), 7.15 (s, 1H), 6.33 (s, 2H, CH_2_), 2.41 (s, 3H, CH_3_ ), 2.35 (s, 3H, CH_3_). ^13^C-NMR (CDCl_3_) δ 150.8, 148.6, 141.5, 136.8, 135.6, 135.3, 133.3, 133.1, 132.7, 131.9, 128.4, 127.8, 127.7, 126.2, 125.8, 125.4, 125.2, 124.9, 124.5, 123.6, 120.1, 110.8, 49.1, 20.8, 20.4. IR (KBr) 1440 cm^−1^. Anal. Calcd. For C_25_H_21_N_3_ (MW 363.45): C, 82.61%; H, 5.82%; N, 11.56%. Found: C, 82.97%; H, 6.15%; N, 11.50%.

*1-(Naphthalen-2-ylmethyl)-2-(pyridin-3-yl)-1H-benzo[d]imidazole* (**49**). Yield = 86%. Mp. 146.3–147.0 °C. ^1^H-NMR (CDCl_3_) δ 9.02 (s broad, 1H), 8.74 (s broad, 1H), 8.06 (d, *J* = 7.3 Hz, 1H), 7.97 (d, *J* = 7.6 Hz, 1H), 7.92–7.82 (m, 2H), 7.72 (d, *J* = 7.6 Hz, 1H), 7.57–7.46 (m, 3H), 7.44–7.37 (m, 2H), 7.35–7.24 (m, 3H), 5.65 (s, 2H, CH_2_). ^13^C-NMR (CDCl_3_) δ 150.9, 136.8, 136.3, 135.0, 133.4, 133.3, 133.0, 131.7, 129.3, 127.9, 127.8, 126.8, 126.5, 124.6, 123.8, 123.6, 123.2, 120.3, 114.1, 112.8, 110.7, 110.7, 48.6. IR (KBr) 1459.5 cm^−1^. Anal. Calcd. For C_23_H_17_N_3_ (MW 335.40): C, 82.36%; H, 5.11%; N, 12.53%. Found: C, 81.99%; H, 5.54%; N, 12.55%. 

*5-Fluoro-1-(naphthalen-2-ylmethyl)-2-(pyridin-3-yl)-1H-benzo[d]imidazole* (**50**). Yield = 66%. Mp. 175.7–176.8 °C ^1^H-NMR (CDCl_3_) δ 9.00 (s, 1H), 8.74 (s broad, 1H), 8.05 (d, *J* = 7.8 Hz, 1H), 7.93–7.80 (m, 3H), 7.73 (d, *J* = 7.9 Hz, 1H), 7.56–7.50 (m, 2H), 7.48–7.46 (m, 1H), 7.43–7.37 (m, 1H), 7.26 (d, *J* = 8.5 Hz, 1H), 7.13 (td, *J*_1_ = 9.2 Hz, *J*_2_ = 2.3 Hz, 1H), 6.99 (dd, *J*_1_ = 8.5 Hz, *J*_2_ = 2.4 Hz, 1H), 5.62 (d, *J* = 14.7 Hz, 2H, CH_2_). ^13^C-NMR (CDCl_3_) δ 160.1 (d, *J* = 234.2 Hz), 151.9, 151.1, 149.7, 139.7, 136.6 (d, *J* = 8.2 Hz), 133.4, 133.0, 132.8, 129.5, 127.9 (d, *J* = 4.8 Hz), 126.8, 126.6, 126.3, 124.6, 123.6, 123.5, 121.2 (d, *J* = 10.1 Hz), 112.3, 111.7 (d, *J* = 25.7 Hz), 105.9, 97.4 (d, *J* = 27.5 Hz), 48.8. IR (KBr) 1478 cm^−1^. Anal. Calcd. For C_23_H_16_FN_3_ (MW 353.39): C, 78.17%; H, 4.56%; N, 11.89%. Found: C, 77.88%; H, 4.96%; N, 11.39%. 

*6-Fluoro-1-(naphthalen-2-ylmethyl)-2-(pyridin-3-yl)-1H-benzo[d]imidazole* (**51**). Yield = 62%. Mp. 166.8–167.4 °C. ^1^H-NMR (CDCl_3_) δ 9.00 (s, 1H), 8.75 (d, *J* = 4.8 Hz, 1H), 8.07 (d, *J* = 8.1 Hz, 1H), 7.94–7.83 (m, 2H), 7.77–7.70 (m, 1H), 7.61 (dd, *J*_1_ = 9.1 Hz, *J*_2_ = 2.4 Hz, 1H),7.56–7.50 (m, 2H), 7.48 (s, 1H), 7.42 (dd, *J*_1_ = 8.0 Hz, *J*_2_ = 4.8 Hz, 1H), 7.31–7.24 (m, 1H), 7.22 (dd, *J*_1_ = 8.8 Hz, *J*_2_ = 4.5 Hz, 1H), 7.06 (td, *J*_1_ = 9.0 Hz, *J*_2_ = 2.3 Hz, 1H), 5.65 (s, 2H, CH_2_).^13^C-NMR (CDCl_3_) δ 159.9 (d, *J* = 238.9 Hz), 151.1, 149.7, 144.4, 143.7, 136.7, 133.4, 133.0, 130.0, 129.4, 129.4, 127.8 (d, *J* = 4.5 Hz), 126.9, 126.6, 125.9, 124.6, 123.5, 118.4, 116.1, 112.2 (d, *J* = 26.3 Hz), 111.1 (d, *J* = 10.2 Hz), 106.0 (d, *J* = 24.3 Hz), 48.8. IR (KBr) 1500 cm^−1^. Anal. Calcd. For C_23_H_16_FN_3_ (MW 353.39): C, 78.17%; H, 4.56%; N, 11.89%. Found: C, 78.30%; H, 4.59%; N, 11.92%.

*5,6-Dimethyl-1-(naphthalen-2-ylmethyl)-2-(pyridin-3-yl)-1H-benzo[d]imidazole* (**52**). Yield = 96%. Mp. 138.5–139.5 °C. ^1^H-NMR (CDCl_3_) δ 8.99 (s broad, 1H), 8.70 (s broad, 1H), 8.03 (d, *J* = 7.5 Hz, 1H), 7.94–7.82 (m, 2H), 7.77–7.66 (m, 2H), 7.56–7.44 (m, 3H), 7.36 (s broad, 1H), 7.29 (d, *J* = 6.5 Hz, 1H), 7.08 (s, 1H), 5.60 (s, 2H, CH_2_), 2.44 (s, 3H, CH_3_ ), 2.35 (s, 3H, CH_3_ ). ^13^C-NMR (CDCl_3_) δ 150.7, 142.0, 136.5, 135.0, 133.7, 133.5, 133.1, 132.9, 132.2, 129.3, 127.9, 127.8, 126.7, 126.4, 124.5, 123.6, 123.5, 120.3, 110.6, 107.1, 103.8, 103.0, 48.5, 20.6, 20.4. IR (KBr) 1447 cm^−1^. Anal. Calcd. For C_25_H_21_N_3_ (MW 363.45): C, 82.61%; H, 5.82%; N, 11.56%. Found: C, 82.66%; H, 5.92%; N, 11.66%. 

*1-(Naphthalen-2-ylmethyl)-2-(pyridin-4-yl)-1H-benzo[d]imidazole* (**53**). Yield = 94%. Mp. 133.0-131.0 °C. ^1^H-NMR (CDCl_3_) δ 8.73 (d, *J* = 5.9 Hz, 2H), 7.97 (d, *J* = 8.1 Hz, 1H), 7.94–7.86 (m, 2H), 7.73 (d, *J* = 6.6 Hz, 1H), 7.67 (d, *J* = 5.9 Hz, 2H), 7.58–7.48 (m, 3H) 7.45–7.37 (m, 1H), 7.36–7.28 (m, 3H), 5.68 (s, 2H, CH_2_). ^13^C-NMR (CDCl_3_) δ 150.5(2C), 143.2, 137.7, 136.5, 133.3, 129.4, 127.9, 127.8, 126.8, 126.5, 124.6, 124.2, 123.5, 123.4, 123.2(2C), 120.6, 119.5, 119.3, 118.7, 110.7, 48.7. IR (KBr) 1437 cm^−1^. Anal. Calcd. For C_23_H_17_N_3_ (MW 335.40): C, 82.36%; H, 5.11%; N, 12.53%. Found: C, 81.22%; H, 5.60%; N, 12.68%.

*5-Fluoro-1-(naphthalen-2-ylmethyl)-2-(pyridin-4-yl)-1H-benzo[d]imidazole* (**54**). Yield = 50%. Mp. 177.8–178.5 °C. ^1^H-NMR (CDCl_3_) δ 8.76 (s broad, 2H), 7.95–7.85 (m, 2H), 7.77–7.71 (m, 1H), 7.67 (s broad, 2H), 7.62 (dd, *J*_1_ = 9.2 Hz, *J*_2_ = 2.4 Hz, 1H), 7.58–7.51 (m, 2H), 7.48 (s, 1H), 7.32–7.26 (m, 1H), 7.22 (dd, *J*_1_ = 8.8 Hz, *J*_2_ = 4.4 Hz, 1H), 7.06 (td, *J*_1_ = 9.1 Hz, *J*_2_ = 2.4 Hz, 1H), 5.66 (s, 2H, CH_2_). ^13^C-NMR (CDCl_3_) δ 158.7, 151.5 (d, *J* = 214.6 Hz), 143.5, 138.7, 137.4, 133.3 (d, *J* = 12.0 Hz), 133.0, 132.9(2C), 131.7, 129.5, 127.8(2C), 127.7, 126.9, 126.6, 125.1, 124.6, 123.4, 119.1, 112.6 (d, *J* = 26.5 Hz), 111.2 (d, *J* = 11.0 Hz), 106.2 (d, *J* = 24.3 Hz), 48.8. IR (KBr) 1585 cm^−1^. Anal. Calcd. For C_23_H_16_FN_3_ (MW 353.39): C, 78.17%; H, 4.56%; N, 11.89%. Found: C, 78.66%; H, 4.57%; N, 11.79%. 

*6-Fluoro-1-(naphthalen-2-ylmethyl)-2-(pyridin-4-yl)-1H-benzo[d]imidazole* (**55**). Yield = 53%. Mp. 163.5–164.1 °C. ^1^H-NMR (CDCl_3_) δ 8.79 (s broad, 2H), 8.00–7.82 (m, 3H), 7.80–7.60 (m, 2H), 7.59–7.44 (m, 3H), 7.37–7.25 (m, 2H), 7.15 (td, *J*_1_ = 9.2 Hz, *J*_2_ = 2.4 Hz, 1H), 6.99 (dd, *J*_1_ = 8.5 Hz, *J*_2_ = 2.4 Hz, 1H), 5.64 (s, 2H, CH_2_). ^13^C-NMR (CDCl_3_) δ 153.3 (d, *J* = 227.6 Hz), 150.4, 147.3, 144.0, 136.0, 134.2, 133.4, 132.7, 132.7, 129.6, 127.9(2C), 126.9, 126.6, 124.6, 124.1, 123.4(2C), 121.5 (d, *J* = 8.1 Hz), 120.5, 112.0 (d, *J* = 23.3 Hz), 97.4 (d, *J* = 26.9 Hz), 48.9. IR (KBr) 1497 cm^−1^. Anal. Calcd. For C_23_H_16_FN_3_ (MW 353.39): C, 78.17%; H, 4.56%; N, 11.89%. Found: C, 78.18%; H, 4.99%; N, 11.55%.

*5,6-Dimethyl-1-(naphthalen-2-ylmethyl)-2-(pyridin-4-yl)-1H-benzo[d]imidazole* (**56**). Yield = 90%. Mp. 133.7-134.5 °C. ^1^H-NMR (CDCl_3_) δ 8.69 (d, *J* = 6.0 Hz, 2H), 7.96–7.85 (m, 2H), 7.77–7.69 (m, 2H), 7.64 (d, *J* = 6.0 Hz, 2H), 7.58–7.51 (m, 2H), 7.49 (s, 1H), 7.33 (d, *J* = 8.5 Hz, 1H), 7.08 (s, 1H), 5.64 (s, 2H, CH_2_), 2.44 (s, 3H, CH_3_ ), 2.35 (s, 3H, CH_3_ ). ^13^C-NMR (CDCl_3_) δ 162.2, 161.3, 150.5, 150.4(2C), 142.2, 133.7, 129.4, 127.9, 127.8, 126.8, 126.5, 126.5, 124.8, 124.4, 123.6, 123.1(2C), 120.4, 115.5, 112.8, 110.7, 109.1, 96.5, 50.2. IR (KBr) 1450 cm^−1^. Anal. Calcd. For C_25_H_21_N_3_ (MW 363.45): C, 82.61%; H, 5.82%; N, 11.56%. Found: C, 83.06%; H, 5.88%; N, 11.78%.

### 3.2. Pharmacological Evaluation Binding Assays

For the CB1 receptor binding assays the new compounds were first subjected to a preliminary screen carried out using three concentrations of test compounds (1, 0.1 and 0.01 µM), human cannabinoid CB1 receptor (PerkinElmer Inc., Waltham, MA, USA, Product No.: 6110129400UA, Lot No.: 509–845-A) and [^3^H]-(-)-*cis*-3-[2-hydroxy-4-(1,1-dimethylheptyl)phenyl]-*trans*-4-(3-hydroxy-propyl)cyclohexanol ([^3^H]CP-55,940; Kd = 500 pM) as the high affinity ligand as described by the manufacturer (Perkin-Elmer). Compounds which displaced [^3^H]CP-55,940 by more than 50% at 1 µM were further analyzed. Displacement curves were generated by incubating drugs with 0.5 nM of [^3^H]CP-55,940. In all cases, K_i_ values were calculated by applying the Cheng-Prusoff equation [34] to the IC_50_ values (obtained by GraphPad, GraphPad Software, Inc., La Jolla, CA, USA) for the displacement of the bound radioligand by increasing concentrations of the test compounds (after three separate experiments for each compound).

### 3.3. Construction of the hCB1 Receptor Homology Model

The sequence of the human CB1 receptor was retrieved from UniProt database with accession number P21554. The crystal structure of human adenosine A_2A_ receptor (PDB ID: 3EML) [35], was identified from the Protein Data Bank (PDB) and selected as the template for homology modeling according to the results of a BLAST search. The CB1R model was generated by comparative modeling using MODELLER v9.10 [36,37]. The selection of the template was reinforced with evidence from molecular phylogenetic reports that indicate that this receptor is more evolutionarily related to CB1 than the other members of family A GPCRs for which a crystal structure exists. The Clustal obtained alignment was manually modified to preserve functionally important residues and the disulfide bridge between residues C257 and C264. The residues corresponding to segment 306–324 of intracellular loop 3 were not considered in the modeling. The final alignment obtained between both sequences presents 25.4% and 49.2% of sequence identity and similarity, respectively. Most conserved residues within each helix are denoted using the Ballesteros-Weinstein notation [38]. A set of 100 models was generated, and the best model according to the internal scoring function of the program (PDF score) was subjected to an energy minimization protocol in order to relax the structure and optimize bond geometry. Model validation was performed using the tools available at the NIH SAVES server (http://nihserver.mbi.ucla.edu/SAVES/).

**Figure 9:**
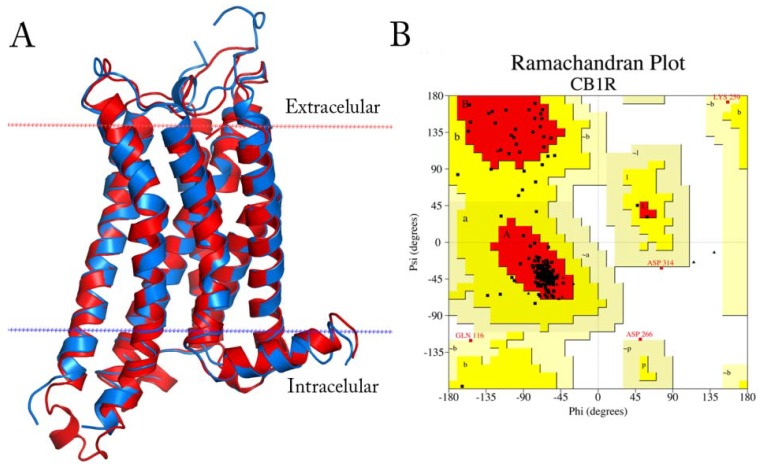
(**A**) Superimposition of the obtained CB1R model (red) with the A_2A_R template (blue). The plasma membrane is delimited by the red and blue dotted lines (**B**) Ramachandran plot for the CB1R model.

The model presents an alpha-carbon root-mean square deviation of 0.64 Å against the template structure (Figure 9A). The final model contains residues 113–420, with a disulfide bridge between C257 and C264, and presents more than 97% of the residues in allowed regions according to Ramachandran plot analysis [39] (Figure 9B).

### 3.4. Docking of the Library of Compounds

Molecular docking of the compounds was performed using FRED v2.2.5 (OpenEye Scientific Software, Santa Fe, NM, USA). The CB1 model binding site was prepared using the FRED receptor, considering the residues involved in the interaction of CB1 with WIN55212, as our group has reported previously [24]. A multiconformer library was prepared using OMEGA v2.4.3 (OpenEye Scientific Software). Candidate poses of the ligands within the receptor site (100) were obtained and optimized using the Chemgauss3 scoring function. Consensus structures of the poses returned from exhaustive docking and optimization were obtained by consensus scoring using the PLP, Chemscore and Chemgauss3 scoring functions. Finally, the top ranked binding modes for each compound were minimized using the CHARMM22 force-field in Discovery Studio v2.1 (Accelrys Inc., San Diego, CA, USA). The minimization protocol allows movement of the side chains of residues positioning within a radius of 6 Å from the mass centroid of all docked ligands, using the conjugate gradient algorithm to a convergence criterion of 0.001 kcal/mol Å for the RMS of energy gradient. Energy evaluation was performed for each obtained complex using the PLP, LigScore, PMF, and LUDI scoring functions. Additional binding energy calculations were performed using the PBSA (Poisson Boltzmann with non-polar Surface Area) implicit solvent model [40], with a dielectric constant of 4 in order to simulate the membrane environment for the complexes.

### 3.5. Generation of CoMFA and Partial Least Squares (PLS) Analysis

CoMFA studies were performed using SYBYL-X 1.2 molecular modeling software (Tripos Inc., St. Louis, MO, USA). Models of steric and electrostatic fields for CoMFA were based on both Lennard–Jones and Coulombic potentials [41]. Steric and electrostatic energies were calculated using an sp^3^ carbon atom with a van der Waals radius of 0.152 nm, +1 charge, and 0.2 nm grid spacing. The CoMFA cutoff values were set to 125.46 kJ/mol for both steric and electrostatic fields.

PLS analysis was used to construct a linear correlation between the CoMFA descriptors (independent variables) and the affinity values (dependent variables) [42]. To select the best model, the cross-validation analysis was performed using the LOO method (and SAMPLS), which deduces the square of the cross-validation coefficient (*q^2^*) and the optimal number of components *N*. The non-cross-validation was performed with a column filter value of 2.0 to speed up the analysis and reduce the noise. The predictive *r^2^* (*r^2^_pred_*) [43,44] was based only on molecules not included in the training set and was obtained according to the following equation: *r^2^_pred_* = (SD − PRESS)/SD (Table 2).

## 4. Conclusions

In summary, we have discovered a series of novel and potent CB1 receptor ligands. The benzimidazole derivatives were rationally designed based on chemical modifications of the prototype compound **14**. A major focus of the optimization effort was to increase CB1 affinity.

Biological characterization of these compounds in radioligand binding assays established that the position of the nitrogen atom on the pyridine ring is crucial for activity. Both 3- and 4-pyridyl groups yield active compounds, but 3-pyridyl is a much better substituent than 4-pyridyl and the 2-pyridyl seems useless. These results suggest possible hydrogen bonding with K3.28(192). No significant differences in affinity were observed when the carbonyl linker was replaced by a methylene group.

Changing the position of the naphthyl ring from α to β resulted in a decrease of CB1 receptor affinities. Only two of the most potent derivatives are not substituted on the benzimidazole ring, suggesting that substitution at the C5 and/or C6 positions is beneficial for CB1 affinity, probably by enhancing π-stacking interactions in which F2.57(170), F3.36(200) and W6.48(356) may be involved.

The QSAR model gave good statistical results in terms of *q^2^* and *r^2^* values. Our CoMFA analysis based on binding affinity data of benzimidazole ligands for the CB1 receptor allowed us to approach optimal substitution in the regions labeled I, II and III. The analysis of the contour maps indicates that the activity of the compounds is due largely to their electronic properties rather than to steric or lipophilic contributions, defining two key areas for further chemical modification: the naphthalene and the pyridine rings. The information gained in this work should lead to the development of additional high-affinity CB1 receptor ligands.
